# An innate immune signature induced by AS01- or AS03-adjuvanted vaccines predicts the antibody response magnitude and quality consistently over time

**DOI:** 10.3389/fimmu.2024.1412732

**Published:** 2024-08-14

**Authors:** Setareh Tasdighian, Viviane Bechtold, Ahmed Essaghir, Yvan Saeys, Wivine Burny

**Affiliations:** ^1^ Center for Inflammation Research, VIB, Ghent, Belgium; ^2^ Department of Applied Mathematics, Computer Science and Statistics, Ghent University, Ghent, Belgium; ^3^ GSK, Rixensart, Belgium

**Keywords:** antibody, adjuvant, AS01, AS03, innate immunity, prediction, systems serology

## Abstract

**Background:**

Antibody-mediated protection can depend on mechanisms varying from neutralization to Fc-dependent innate immune-cell recruitment. Adjuvanted vaccine development relies on a holistic understanding of how adjuvants modulate the quantity/titer and quality of the antibody response.

**Methods:**

A Phase 2 trial (ClinicalTrials.gov: NCT00805389) evaluated hepatitis B vaccines formulated with licensed adjuvants (AS01_B_, AS01_E_, AS03, AS04 or Alum) in antigen-naïve adults. The trial investigated the role of adjuvants in shaping antibody-effector functions, and identified an innate transcriptional response shared by AS01_B_, AS01_E_ and AS03. We integrated previously reported data on the innate response (gene expression, cytokine/C-reactive protein levels) and on quantitative/qualitative features of the mature antibody response (Fc-related parameters, immunoglobulin titers, avidity). Associations between the innate and humoral parameters were explored using systems vaccinology and a machine-learning framework.

**Results:**

A dichotomy in responses between AS01/AS03 and AS04/Alum (with the former two contributing most to the association with the humoral response) was observed across all timepoints of this longitudinal study. The consistent patterns over time suggested a similarity in the impacts of the two-dose immunization regimen, year-long interval, and non-adjuvanted antigenic challenge given one year later. An innate signature characterized by interferon pathway-related gene expression and secreted interferon-γ-induced protein 10 and C-reactive protein, which was shared by AS01 and AS03, consistently predicted both the qualitative antibody response features and the titers. The signature also predicted from the antibody response quality, the group of adjuvants from which the administered vaccine was derived.

**Conclusion:**

An innate signature induced by AS01- or AS03-adjuvanted vaccines predicts the antibody response magnitude and quality consistently over time.

## Introduction

The identification of parameters influencing the immune response to vaccination can inform the development of more effective and personalized vaccination strategies. Historically the humoral vaccine response has been assessed based on neutralizing antibody (nAb) titers or antigen-binding immunoglobulin (Ig)G titers, which often intercorrelate (1). Across different vaccines, these metrics are however inconsistently associated with antibody-mediated protection, likely because they do not reflect the full complexity of the polyclonal antibody response to vaccination. In addition to IgG titers, titers of IgM, IgA and IgG/IgA subclasses may play a role, and can also correlate with total IgG or nAb titers (1). Beyond binding and neutralization, antibodies with Fc-dependent functions such as Fcγ-receptor (FCGR)-binding antibodies, as well as antibody-dependent (AD) innate immune functions, e.g., AD cellular phagocytosis (ADCP) (2, 3), can be important. Indeed, such features, which are typically determined in systems serology studies, have been shown to contribute to immunity against various infectious diseases, including influenza, malaria, and respiratory syncytial virus (RSV) disease, as well as against certain types of cancer (3–8). Another functional measure is antibody avidity, which reflects the strength of the interaction between antibodies and their epitopes (9). Though less is known about the avidity of different antibody isotypes, IgG avidity was associated with viral neutralization in infections with severe acute respiratory syndrome coronavirus 2 (SARS-CoV-2), RSV, cytomegalovirus, varicella zoster virus, and human immunodeficiency virus (1, 10–15). Finally, as waning immunity may necessitate boosters, the persistence of the antibody response is a critical determinant of the vaccination regimen to be applied.

By activating innate immunity, vaccine adjuvants can shape the immunogenicity of a vaccine by promoting antibody production and immune cell activation (16). Indeed, adjuvants can improve nAb or binding titers, antibody avidity and response persistence, as well as antibody-mediated functions/FcR activity (17–22) as shown for the malaria vaccine containing Adjuvant System (AS)01 (8). AS01 is a liposomal adjuvant containing the Toll-like receptor (TLR) 4 agonist 3-*O*-desacyl-4′-monophosphoryl lipid A (MPL) and the saponin QS-21. The molecular and cellular mechanisms underlying such immune enhancements can be unraveled in systems vaccinology studies (23, 24). This was done for example in an adjuvanted influenza vaccine study, which identified innate and adaptative immune signatures that correlated with the antibody response persistence (25). Furthermore, AS01_B_, AS01_E_ (half-dose AS01_B_ with respect to MPL and QS-21 quantities), AS03 (oil-in-water emulsion with α-tocopherol), AS04 (containing MPL adsorbed on aluminum salt [Alum]), and Alum, each combined with hepatitis B surface antigen (HBsAg), have been compared with respect to their abilities to improve various aspects of the innate and adaptive immune response, in a trial in HBsAg-naïve young adults (NCT00805389). Indeed, in the multiple reports on this single clinical trial, we characterized the innate cellular, cytokine and transcriptional responses, the T-cell and memory B-cell responses, the antibody titers, avidity, and response persistence, and the systems serology parameters (21, 26–29). The peak titers after two vaccinations were shown to be associated with the innate immune response, which was for each AS driven by increased serum interleukin (IL)-6 and C-reactive protein (CRP) levels (26, 27). For AS01 and AS03, the association was also governed by parameters of the interferon (IFN)-signaling pathway, centering around a core innate gene signature characterized by upregulation of IFN and innate cell-related genes and downregulation of natural killer (NK) cell-associated genes (26, 27). Thus, the data deeply characterized the immune mechanisms of these AS, which are all present in licensed vaccines. These vaccines include the herpes zoster vaccine, the *Plasmodium falciparum* malaria vaccine, and the RSV prefusion-F-protein-based vaccine (for AS01), the (pre)pandemic influenza and SARS-CoV-2 vaccines (for AS03), as well as the vaccine against hepatitis B virus, or HBV, and the bivalent human papillomavirus vaccine (for AS04). However, neither the reports of the current clinical trial nor those of any other adjuvanted vaccine studies provided a holistic view of the associations within the multifaceted datasets that were generated. Moreover, they did not determine whether the measured parameters could also predict the humoral response at different timepoints.

Here, we used a systems vaccinology approach that integrated the ‘omics’ and other datasets from this clinical study to evaluate whether the innate response (represented by transcriptomics data and CRP/cytokine levels) could predict the antibody titers and response persistence, as well as the qualitative features of the antibody response (including total Ig titers, systems serology parameters, and avidity). The assessments of this longitudinal study were therefore performed not only at peak titer, but also at a persistence timepoint after a year-long interval, and after a subsequent non-adjuvanted, fractional-dose antigenic challenge (21, 29). We identified an innate immune signature that consistently predicted the quantitative and qualitative antibody response features at the peak, persistence, and post-challenge timepoints. This signature consisted of IFN-pathway-related gene modules, and secreted IFN-γ-induced protein 10 (IP-10) and CRP levels. This was a characteristic of the responses to both the AS01- and AS03-adjuvanted vaccines. When confirmed in subsequent studies, this predictive innate marker can be used to optimize the use of these clinically relevant adjuvants, to ultimately improve the design and development of new and/or existing vaccines.

## Materials and methods

### Study design

**Figure 1:**
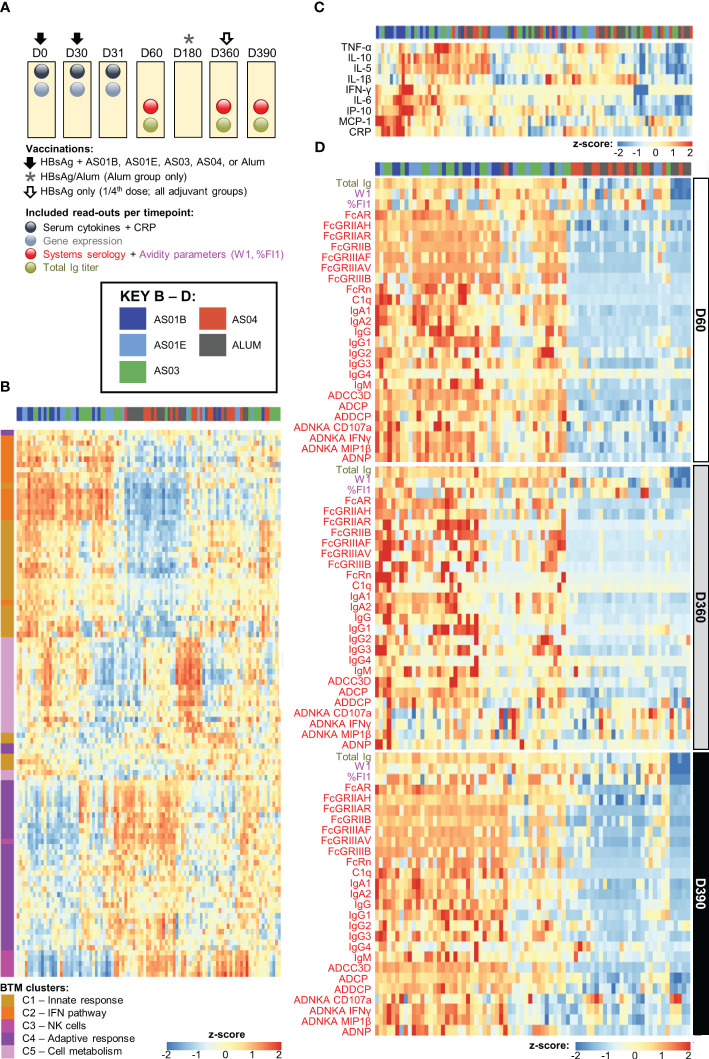
Adjuvant dichotomy in the separate datasets. **(A)** Study design showing vaccination and blood collection timepoints. Participants received two doses of hepatitis B antigen (HBsAg) vaccines adjuvanted with AS01_B_, AS01_E_, AS03, AS04 or Alum at day (D)0 and D30 (and, in the Alum group, a third dose at D360), and an antigenic challenge with non-adjuvanted fractional-dose HBsAg at D360, depicted by the arrows and asterisk. As indicated above the boxes, blood samples were collected at baseline (D0) as well as before and 1 day, 1 month, and 5 months after the second dose of adjuvanted vaccine (D30, D31, D60, D180) and before and 1 month after the challenge (D360 and D390, respectively). **(B–D)** Heatmaps represent the separate datasets post-dose 2: the blood transcriptional model (BTM) clusters C1–5 in log fold-change D31/D30 **(B)**, the serum cytokine and C-reactive protein (CRP) levels in log fold-change D31/D30 **(C)**, and the total immunoglobulin (Ig) titer and systems serology and antibody avidity data at D60, D360 and D390 in log fold-change over D30 **(D)**; see **Table 1** for details and abbreviations. Font colors of the parameters listed left of the heatmaps in **(C, D)** correspond to those of the readouts presented in **(A)**.

This exploratory *post-hoc* analysis was conducted using serum-based data from participants of a Phase II randomized multicenter trial (NCT00805389). The trial protocol was approved by all institutional Ethics Committees and conducted in accordance with the Helsinki Declaration and Good Clinical Practice guidelines. Written informed consent was obtained from each participant before trial participation. The evaluations of the main and secondary endpoints of this trial have been reported previously (21, 26–29). The 18 to 45-year-old, HBV-naïve male or female trial participants were immunized intramuscularly with 20 μg HBsAg adjuvanted with AS01_B_ (n = 15), AS01_E_ (n = 19), AS03 (n = 25), AS04 (*Fendrix*; n = 19), or Alum (*Engerix-B*; n = 20), on day (D)0 and D30. AS01_B_ is an Adjuvant System containing MPL, QS-21 (*Quillaja saponaria* Molina, fraction 21; licensed by GSK from Antigenics LLC., a wholly owned subsidiary of Agenus Inc., a Delaware, USA corporation) and liposome (50 μg MPL and 50 μg QS-21). AS01_E_ is an Adjuvant System containing MPL, QS-21 and liposome (25 μg MPL and 25 μg QS-21). AS03 is an Adjuvant System containing DL-α-tocopherol and squalene in an o/w emulsion. AS04 is an Adjuvant System containing MPL (50 μg) adsorbed on aluminum salt (500 μg Al^3+^). On D360, the participants were revaccinated intramuscularly with a non-adjuvanted fractional dose (5 μg HBsAg). As described (21, 26–29), serum samples were collected before the first dose, second dose or booster (D0, D30 or D360, respectively), at one day, one month and six months post-dose two (D31, D60 and D180) as well as at 1 month post-booster (D390). The serum samples used for the current antibody profiling were collected on D60, D360, and D390; see **Figure 1A**; **Table 1**. Data from 282 anonymized participants who received the booster on D360 were filtered to include only the 98 participants for whom microarray data from peripheral blood mononuclear cells (PBMCs) were available for both D30 and D31.

**Table 1:** Innate and antibody response input parameters.

Class	Category	Parameters		Unit	Timepoints included	Ref.
		N	Description			
Innate response	Cytokines	8	IFN-γ, TNF-α, IP-10, MCP-1, IL-1β, IL-5, IL-6, IL-10	fg/mL	D0, D30, D31	(26)
	Hematology	1	C-reactive protein	mg/dL	D0, D30, D31	(26)
	Microarrays	2,445	Expression of unique genes	Log FC	D0, D30, D31	(27)
Humoral response	Antibody titer	1	Total Ig (by chemiluminescent immunoassay)	MIU/mL	D60, D360, D390	(29)
	Antibody avidity	2	–W1 (anti-HBsAg mAb affinity) –%FI1 (anti-HBsAg mAb/total antibodies)	Arbitrary %	D60, D360, D390	(29)
					D60, D360, D390	
	Systems serology	8	Ig Isotypes and subclasses: IgA1, IgA2, IgG, IgG1, IgG2, IgG3, IgG4, IgM	log_10_ MFI	D60, D360, D390	(21)
		9	Fc-binding protein arrays: FcGRIIAH, FcGRIIAR, FcGRIIB, FcGRIIIAF, FcGRIIIAV, FcGRIIIB, FcRn, FcAR, C1q	log_10_ MFI	D60, D360, D390	
		15	Antibody dependent (AD) functions: ADCC3D	log_10_ MFI	D60, D360, D390	
			ADCP, ADDCP, ADNP	phag.score		
			ADDCP_IFNA2, ADDCP_IL10, ADDCP_IL12B, ADDCP_IL1B, ADDCP_IL6, ADDCP_IL8, ADDCP_IP-10, ADDCP_TNF	pg/L		
			ADNK_CD107a, ADNK_IFNG, ADNK_MIP1B	%		

N, number. D, day. IFN-γ, interferon-γ. TNF-α, tumor necrosis factor-α. IP-10, IFN-γ-inducible protein-10, MCP-1, monocyte chemoattractant protein-1, IL-1β/5/6/10, interleukin-1β/5/6/10. mAb, monoclonal antibody. MFI, mean fluorescence intensity. Phag. Phagocytic. Ig, immunoglobulin. MIU/mL, milli-international unit/mL. FC, fold-change. W1, avidity of the first-component (high-avidity) antibodies. %FI1, relative quantity/abundance of the first-component antibodies, defined as the ratio between the quantity of specific antibodies of the first component over the total amount of specific antibodies represented by the total fluorescence intensity of the signal. Fc-binding protein arrays: abilities of HBsAg-specific antibodies to bind to activating or inhibitory Fc gamma receptors (FcGRs), i.e. FcGRIIAH, FcGRIIAR, FcGRIIB, FcGRIIIAF, FCGRIIIAV, and FcGRIIIB, or to FcRn, FcAR, or complement C1q. ADDC3D, antibody-dependent complement C3 deposition. ADCP, antibody-dependent cellular phagocytosis by THP-1 cells. ADDCP, antibody-dependent phagocytosis by monocyte-derived dendritic cells. ADNP, antibody-dependent neutrophil phagocytosis. ADDCP-IFNA2/IL10/IL12B/IL1B/IL6/IL8/IP10/TNF, ADDCP testing positive for levels of cytokines (IFN-α2, IL-12β, IL-1β, IL-6, IL-8 and TNF-α) in multiplexed bead-based assays. ADNKA_CD107a/IFNG/MIP1B, antibody-dependent natural killer (NK)-cell activation with percentage of cells that are positive for activation markers CD107a, IFN-γ, or MIP-1β.

### Data cleaning

Analyses were performed using the datasets for gene expression microarrays, serum cytokine levels, CRP levels, total Ig titers (by chemiluminescent immunoassay), scores for 32 systems serology features, and anti-HBsAg mAb avidity (W1 and %FI_1_ (29); see **Table 1** for data sources, parameter descriptions, abbreviations, and unit measurements. The QC’d, GCRMA log-transformed microarray dataset of 54,675 unique probe-set IDs (27) was cleaned by (i) updating microarray data using the HGU133-plus2 annotation (R package hgu133plus2.db), where the biomaRt (R package) annotations were used for the remaining probeset IDs not mapping to any gene name; (ii) removing non-annotated duplicates and discarding the probeset IDs not mapping to a gene symbol; (iii) eliminating the 44,585 probeset IDs with an interquartile range (IQR) ≤ 0.75, a filtering step that did not impair the BTM-based biological interpretation of the data (**Supplementary Figure 1**); and (iv) selecting the probeset IDs with maximum values of the average gene expression for all participants from the probeset IDs mapping to the same gene symbol. This analysis pipeline left 2,445 unique genes in the final dataset (see **Supplementary Figure 2**).

Cytokine data included serum levels of tumor necrosis factor (TNF)-α, IFN-γ-induced protein (IP-10), monocyte chemotactic protein (MCP)-1, interleukin (IL)-1β, IL-5, IL-6, IL-10, and IFN-γ, as described previously (26). IL-1β and IL-6 levels and total Ig titers below the respective assay cut-offs (0.822 pg/mL, 0.822 pg/mL, and 6.2 mIU/mL, respectively) were replaced by a random value drawn from a normal distribution, with a mean of half the threshold and a standard deviation of 1, whereby the distribution was truncated at a minimum of 1 and a maximum equal to the threshold. Systems serology parameters and associated scores were included as described (21).

### Data transformation, integration, normalization, visualization, and significance testing

Log_10_ scaling was applied to the systems serology and total Ig data in all analyses. Log_10_ scaling was also applied to the avidity data in all analyses except the predictive modelling, in which W1 data were classified into high-avidity (W1<5) or low-affinity (W1≥5) classes and (where indicated) the data were reverse-scaled (i.e., multiplied by -1). CRP and cytokine data were log_2_ scaled to align with the microarray data. Heatmaps were based on z-score normalized data using the *scale* function in R. For all analyses including the innate response data (in log fold-change D31/D30) and antibody response data on D60, D360, D390, the following steps were performed. First, blood transcriptional module (BTM) enrichment scores were computed based on log fold-change D31/D30 of the gene expression values (see above); then, BTMs and log fold-change D31/D30 data for cytokines and CRP were integrated into a matrix while excluding the participants with missing values for >50% of the innate features. The values of the 32 serology variables, avidity (W1) and total Ig titers on D60 (or D360 or D390) were integrated, and filtered to only include data from participants which were not missing from the innate D31/D30 dataset; then, the innate (D31/D30) and antibody (D60, D360 or D390) data from all included participants were merged, and the integrated data were z-scaled. Missing values were imputed using the K-Nearest-Neighbors (KNN) method (knnImputation package in R) with K = 10. When innate (D31/D30) or antibody response data were used separately, similar steps were followed to integrate the data into a matrix, filter out participants with missing values for more than half of the features, z-scale the data, and impute missing values using the KNN method (K = 10). The separate heatmaps of different data modalities were plotted using the R function *pheatmap* based on Euclidean distance and with ‘Ward.D2’ as clustering method.

BTM enrichment scores were computed from the log fold-change D31/D30 gene expression values using the Gene Set Variation Analysis (GSVA) package in R, publicly available BTMs (30), and the default minimum/maximum gene-set size (5/500). Functional BTM clusters (D31/D30) were defined using K-means clustering with K = 5. The optimal value for the number of clusters was selected using the elbow method (**Supplementary Figure 3**). Previously described cluster definitions (27) were applied to obtain the five BTM clusters, which related to either the early response parameters (C1), IFN pathway and antiviral sensing (C2), NK cell functions (C3), antibody response and cell division functions (C4), or to metabolism, cell cycle, heme biosynthesis, platelet and erythrocyte functions (C5). BTMs and genes (log fold-change D31/D30) with Pearson correlation values >0.8 were clustered based on hierarchical clustering using the ‘Ward.D2’ distance method. For each gene cluster, the gene with the maximum IQR was selected as representative (see **Supplementary Table 1**, column ‘Cluster_representative’) and used to visualize the top-ranking predictive genes per antibody response feature. These BTM and gene clusters were only used in the post-processing of the results of the predictive modelling.

To perform principal component analysis (PCA) of the integrated innate (D31/D30) and antibody response (D60, D360 and D390) data, the data was first integrated as described above, then PCA was performed using *prcomp* function in R. The R package *factoextra* was used to generate the PCA plots.

Significant differences between adjuvant groups based on BTMs (log change D31/D30) were computed using the *compare_means* function of the R package *ggpubr*, the default non-parametric method (Wilcoxon test), Benjamini-Hochberg (BH)-adjusted p-values, and α = 0.05 as significance threshold. The analysis was repeated using log fold-change D31/D30 values of genes, CRP and cytokines; see **Supplementary Table 2** for variables significantly different between two adjuvant groups, the associated p-values, and the group(s) with the highest average median value (‘adjuvant_Highest_Average_Median’ column).

To obtain the final D31/D30 BTM selection for the adjuvant classification using LASSO and PLSDA (**Supplementary Table 3**), a previously described pipeline (21) was extended to the other data modalities. Following this pipeline, participants with >50% missing values across the feature space were excluded, and any remaining missing values were imputed using KNN with K = 10.

### Predictive modeling

The innate immune variables (log fold-change D31/D30) were used as input to predict 33 antibody response variables on D60, D360 and D390. At each timepoint, the predictive model was run twice: once using the integrated values of log_2_ fold-change D31/D30 values for CRP, cytokines, and BTMs together, and then using the integrated values of log_2_ fold-change D31/D30 values for CRP, cytokines, and gene expression together. Data were split using the common 80:20 ratio (i.e., 80% to train the model; 20% kept unseen to test the model performance). A nested cross-validation approach was used, where the inner loop consisted of 10 repeats of a five-fold cross-validation of the LASSO model on the training set, to perform predictions on the test set. At each repeat, λ (a LASSO regularization parameter) was selected, which corresponded to the average minimum cross-validated Mean Square Error (MSE) over all 10 runs. In the outer loop, the process was repeated for 100 randomly selected train/test sets with the 80:20 ratio. For each of these 100 sets, a Random Forest (RF) model was also fitted, by setting the number of trees to 1000, and optimizing the hyperparameter corresponding to the number of variables to randomly sample candidates at each split (i.e., minimizing the out-of-bag error). R^2^ scores of the antibody response predictions on D60, D360 and D390 were calculated using LASSO and RF, and R^2^ distributions were plotted using the R packages *glmnet* and *randomforest* for LASSO and RF respectively (see **Supplementary Tables 4**, **5** for BTMs and genes, respectively).

For LASSO-based predictive modeling, the model selected a set of input variables (CRP, cytokines, BTMs or genes) that were required to fit a regularized linear regression to the data. Outputs included the deviance ratio (i.e., the model’s superiority with respect to explaining the data relative to the null/intercept model), the LASSO_R^2^ (i.e., the average R^2^ of the fit using the model based on the selected features), and the LASSO_ λ (i.e., the optimal λ over all runs of the nested cross-validation). Models with a deviance ratio <50% were filtered out. For RF-based modeling, the number of trees was set at 1000, and the *mtry* parameter (number of features to consider at each split point) were optimized using the *tuneRF* function of the *randomForest* package in R (with the step-factor parameter set at 1.5 and the ‘improve’ parameter at 0.01). Results were computed using the Average Increase in MSE (%IncMSE), indicating the amount of increase in the MSE when the feature would be randomly permutated (note that the higher the value, the more important the input variable). Models with an average percentage of the explained variance <50% were filtered out. For avidity predictions, W1 values were assigned to either the high-affinity (W1<5) or low-affinity (W1≥5) classes using LASSO and RF classification methods, because the above modeling techniques did not allow predicting continuous W1 values.

### Predictive performance and model accuracy

A binary predictor score (Y/N) was assigned to each antibody response feature on D60, D360 or D390, based on the variance captured by the model (i.e., null-deviance ratio for regularized regression by LASSO; Variance Explained for RF). Only antibody responses explained by >50% using at least one method (LASSO and/or RF) were included in further analyses. The remaining responses were excluded from the postprocessing steps. **Supplementary Tables 4**, **5** present the variances explained by the model (R^2^) using BTMs/genes as input, and the accuracies of the unseen test data in predicting the antibody responses on the three time-points using LASSO and RF. For W1 predictions using BTMs/genes, see **Supplementary Tables 6**, **7**, respectively for the confusion matrix statistics, whereby models with a balanced accuracy ≤ 50% were filtered out, and the feature importance was reported as “mean decrease in accuracy” per feature (indicating the average decrease in MSE when the feature would be randomly permutated).

### Postprocessing of the predictive modelling results

Before reporting the final predictive modeling results, the following post-processing steps were performed:

#### Corrections for correlations in predictors

In the presence of large correlations between the predictors (input variables/innate response), results of the predictive models may be biased towards either selecting only one of the many highly correlated variables (for LASSO), or dividing the importance scores between these variables (for RF). Therefore, log fold-change D31/D30 values of BTMs/genes were clustered using hierarchical clustering, and clusters of BTMs/genes that were >80% correlated (Pearson correlation) across all participants were identified (see above). The method outputs were then corrected as follows. For LASSO, the “count” values for all features within a cluster were averaged, and each feature was assigned an average count instead of the raw count output by the method. For RF, the %IncMSE values for all features in a cluster were averaged, and each feature was assigned the computed average value as the new feature-importance (note that for W1, values of ‘mean decrease in accuracy’ rather than of %IncMSE were used).

#### Ranking features for the prediction of each antibody response feature

For antibody response features on D60, D360 or D390, BTMs/genes were ranked based on their feature importance in the predictive model, using the *dense_rank* function from the R package *dplyr* while allowing ties in the ranking. Next, rankings of BTMs/genes from both LASSO and RF were averaged, and used to present a list and heatmap of the ranked BTMs/genes per response feature, and then the combined rank of LASSO and RF was standardized per feature. Spearman correlations between the rankings of BTMs, CRP and cytokines to predict each antibody response feature (using LASSO and RF on each timepoint) were reported, and correlations between the two rankings were assessed (see **Supplementary Table 8**). For the predictable antibody features, the determined correlations between the two ranking methods were considered satisfactory across the timepoints (predominantly ≥50% for the BTM- or gene-based predictors, and 58% [BTM-based] on both D60 and D360 for all predictable antibody features combined).

### GO enrichment analysis of genes in the top-ranked list

For each predictable antibody response variable on D60, D360 and D390, GO enrichment scores were calculated for the top 5 genes identified based on the average ranks described above, using the *g:GOSt* algorithm from the R package *goprofiler* (*hsapiens* setting with exclusion of Inferred from Electronic Annotation). Medians of the term_size (i.e., the number of genes that are annotated to the term) were computed for all enriched GO terms. Only categories with sizes below the median size were reported (**Supplementary Table 9**). Similar steps were followed for the GO enrichment analysis of the top 10 genes, as presented in **Supplementary Table 10**.

## Results

Our analyses integrated the longitudinal datasets of the innate and adaptive response features induced by the HBsAg vaccines adjuvanted with AS01_B_, AS01_E_, AS03, AS04 or Alum (21, 26–29); see **Table 1**. Responses were measured at baseline (D0), before dose two (D30), at the innate response peak 1 day later (D31), at peak antibody titer (1 month post-dose 2; D60), at D360 (a proxy for the persistence of the antibody response), and 1 month after the non-adjuvanted antigenic challenge (D390; **Figure 1A**). Analyses focused on the innate response after the second vaccination, given its statistical association with the peak antibody titers, while for the innate response after the first vaccination, such association was either found to a lesser degree (for CRP and cytokine responses) or not at all (for gene expression levels) (26, 27). The model input comprised the serum levels of eight cytokines and CRP, the transcriptional response for 2,445 unique genes from PBMCs, and, for the antibody response, the total Ig titer, the avidity parameter W1 (with higher values corresponding to higher avidity (29)), the proportion of high-avidity antibodies %FI_1_ (see footnote **Table 1** for details), as well as the 32 measured system serology features, which were previously shown to intercorrelate (21). Participant data were randomly assigned to either a training dataset or the test dataset, using a nested cross-validation scheme to ensure that the assignments did not bias the results (see Materials and Methods for details on the predictive model). Analysis pipelines are presented in **Supplementary Figure 2**.

### Adjuvant group dichotomy is consistent across innate and antibody response features

Previously we observed a correlation between the expression of specific gene subsets post-dose 2 (D31/D30 contrast) and the peak antibody titers, as well as a dichotomy in the peak (D31/D30 log fold-change) innate responses between AS01/AS03 vs AS04/Alum (27). Here, we aimed to confirm the presence of a similar pattern in the current separate datasets obtained after the second vaccination. First, we used the IQR-filtered log fold-change D31/D30 expression values to compute enrichment scores of 103 predefined (30) functional BTMs (**Supplementary Figure 1**; **Supplementary Table 11**). Following K-means clustering (**Supplementary Figure 3**), the patterns of the obtained functional BTM clusters (C1–5) confirmed the presence of the same adjuvant group dichotomy as was previously discerned in the peak gene expression (**Figure 1B**). Indeed, of the two cluster categories that emerged, one consisted of BTMs related to the innate response (C1), the IFN-pathway (C2), and, to a lesser extent, to cell metabolism (C5). These enrichments were mostly positive for AS01 and AS03 and absent or negative for AS04 and Alum. A second category contained clusters corresponding to functions related to NK cells (C3) and the adaptive response (C4), with enrichments that were mainly negative for AS01/AS03. The C3/C4 data aligned with the preceding analyses, and likely reflected NK cell–T cell interactions prior to NK-cell trafficking from the blood to local sites, which may only occur for AS01/AS03 (27). A similar separation between the adjuvants was detected in the cytokine and CRP levels (log fold-change D31/D30; **Figure 1C**). Taken together, this suggested that the factors governing a robust innate response are mainly induced by AS01 and AS03.

The adjuvant group dichotomy was mirrored by the collective quantitative/qualitative features of the mature antibody response at peak, the persistence timepoint, and after the challenge (D60, D360, and D390, respectively). Indeed, relative to AS04/Alum, total Ig titers, most of the system serology features, the magnitudes of high-avidity antibodies (%FI_1_), and the avidity parameter W1 were all higher for AS01/AS03 (**Figure 1D**), which was reflected by the numbers of ‘high-avidity’ participants (**Supplementary Figure 4**). As seen for the peak titers by themselves (21, 29), several of these features were dampened from D60 to D360 and then, at D390, boosted by the non-adjuvanted challenge. This boost underscored the importance of the late revaccination for the induction of persistent functional humoral immunity. Thus, the AS01/AS03–AS04/Alum dichotomy was observed in the separate innate datasets of transcriptional and serum responses, as well as in the timed antibody features. The latter suggests that AS01 and AS03 consistently induce higher titers of functional antibodies after completion of the full two-dose vaccination schedule, regardless of the study timepoint.

### Persistent association between the innate immunity and antibody responses induced by AS01/AS03

We then set out to extend the association with the (mostly IFN-related) gene expression induced by AS01 and AS03 seen for the peak titers (27), to the full antibody dataset. After mapping all possible correlations between the D31/D30 gene expression (aggregated in BTMs) and the longitudinal (D60/D360/D390) antibody response features, we inspected these contributions to the correlations for all features together.

**Figure 2:**
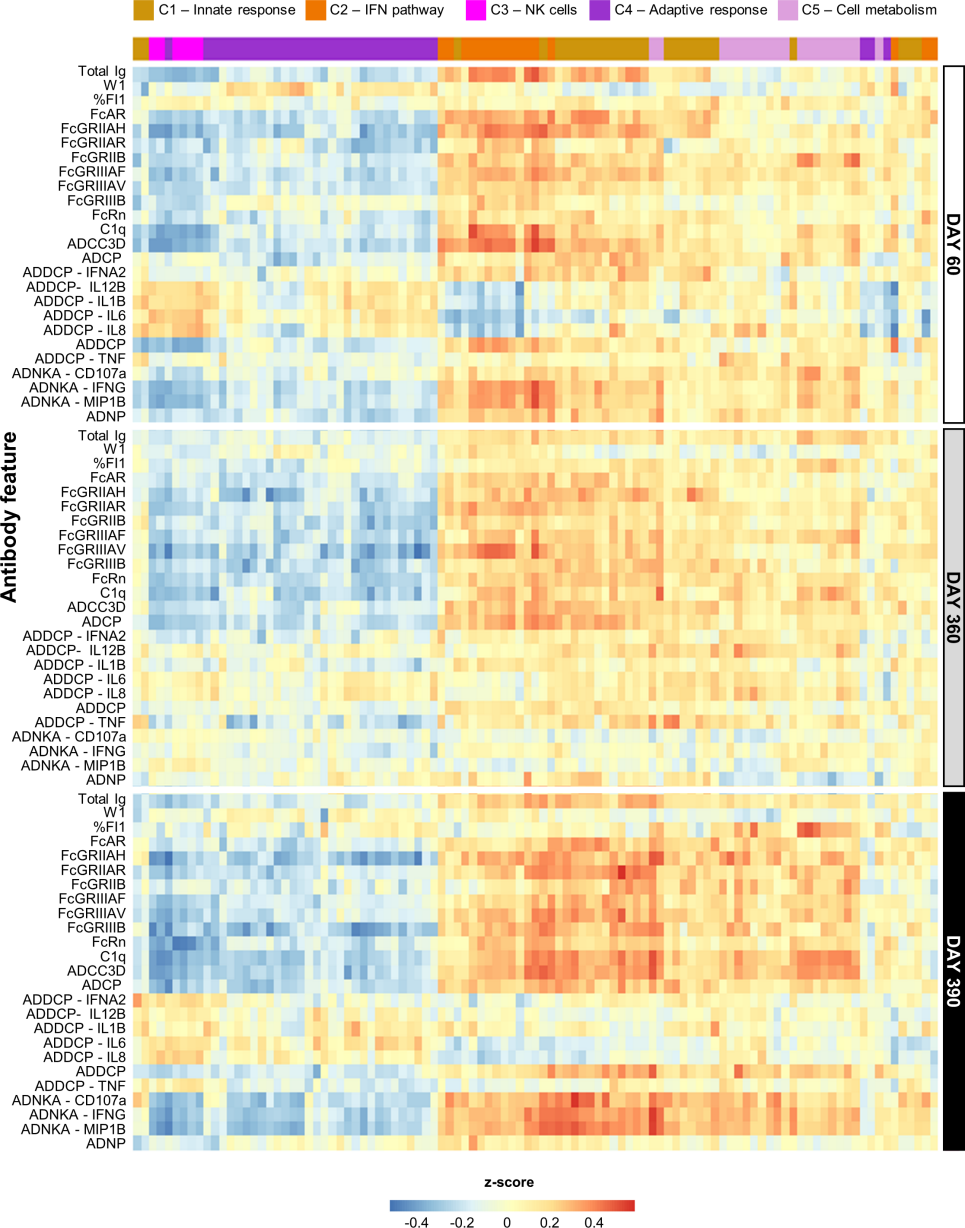
Persistent correlation between innate response and antibody features for AS01 and AS03. Heatmap presents the Pearson correlation between blood transcriptional models (BTMs) and the quantitative and qualitative antibody response parameters (defined in **Table 1**) elicited by the study vaccines adjuvanted with AS01_B_, AS01_E_, or AS03 (see **Supplementary Figure 4** for AS04 and Alum data). BTMs (columns) are presented according to their data-driven clustering into the five functional BTM clusters (C1–C5) color-coded as presented in the key, and antibody response features (rows) are presented by timepoint as depicted right of the heatmap.

Several linear correlations with antibody features were observed for AS01/AS03 (**Figure 2**), which were positive for the innate immunity- and IFN pathway-related clusters (C1 and C2, respectively), and negative for NK-cell-related (C3) and adaptive response-related (C4) functions. Due to the lack of DEGs (27), most BTMs were not enriched for AS04/Alum (**Supplementary Figure 5**), with the exception of several of the C1/C2-related BTMs. The latter BTMs were positively correlated with various Fc-mediated functions, mainly on D60 and D390.

**Figure 3:**
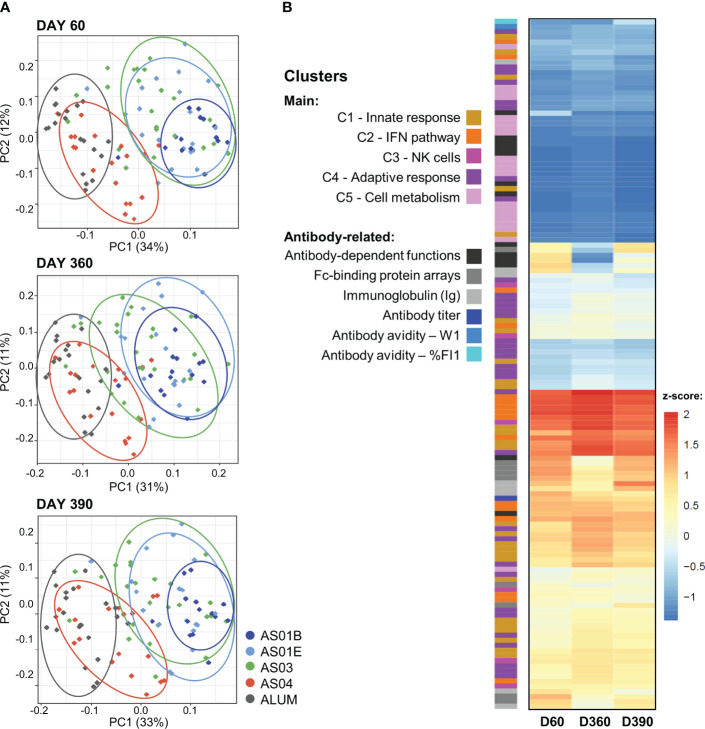
Association between innate and adaptive response features in the PCA-reduced space. **(A)** Principal component (PC) analysis (PCA) of the innate response (in blood transcriptional models; BTMs) and the quantitative and qualitative antibody response features measured on day (D)60 (top), D360 (middle) and D390 (bottom) was performed by participant and treatment group (see **Supplementary Figure 5** for scree plots). The variance explained by the first two PCs (PC1, PC2) is indicated in the brackets along the axes. Each dot represents an individual participant, color-coded by adjuvant group as shown in the key. Arbitrary aggregation of the participants into groups is visualized by the ellipses, according to the color coding presented beside the plots. **(B)** Heatmap represents the contributions of the functional BTM clusters or antibody-related features, color-coded as presented in the key, to the first PC (note that W1 values were multiplied by -1 such that higher values correspond to higher avidity); see **Supplementary Figures 6**, **7** for annotations of individual BTMs of the PC1 and PC2, respectively. Antibody-dependent functions include: ADCC3D, ADCP, ADDCP, ADNP, ADDCP_IFNA2, ADDCP_IL10, ADDCP_IL12B, ADDCP_IL1B, ADDCP_IL6, ADDCP_IL8, ADDCP_IP-10_ADDCP_TNF, ADNK_CD107a, ADNK_IFNG, and ADNK_MIP1B; Fc-binding protein arrays include: FcGRIIAH, FcGRIIAR, FcGRIIB, FcGRIIIAF, FcGRIIIAV, FcGRIIIB, FcRn, FcAR, and C1q (see footnote of **Table 1** for details).

Exploration of these relations between BTMs and the longitudinal antibody response in the PCA-reduced space further confirmed the adjuvant dichotomy (**Figure 3A**). On each timepoint, there was a clear separation between the data for the AS01/AS03 and the AS04/Alum vaccinees detectable in the PC1 dimension, which captured circa one-third of the variation per timepoint (see **Supplementary Figure 6** for scree plots). A heatmap of the timed associations in the PC1 confirmed that the IFN/innate immunity-related pathways (C1/C2) provided the main (positive or negative) contributions, which were remarkably consistent across the peak, persistence, and post-challenge timepoints (**Figure 3B**; see **Supplementary Figure 7** for BTM annotations). Smaller contributions were seen for most of the quantitative and functional antibody response features. For the PC2 (explaining 11–12% of the variation; **Supplementary Figure 5**), metabolism-related BTMs (C5) provided the strongest – and almost exclusively positive – contributions (**Supplementary Figure 8**). These contributions increased over time, consistent with the data in **Figure 2**.

Thus, for AS01 and AS03, the contributions from the innate/IFN-related responses to the correlation with the comprehensive set of the antibody response features were stable across the different phases of the mature antibody response.

### Early innate responses predict both the persistent and the post-booster antibody response

The results thus far underscored the relevance of the D31/D30 innate data for the association with the antibody response, aligned with the strictly transcriptional-based analyses (27). We then aimed to identify the antibody features at the peak, persistence, and post-challenge timepoints that could be *predicted* by the innate data at these timepoints, considering not only BTMs, but also the CRP or cytokine protein data. Linear and non-linear models, which considered the collective data from participants across all five adjuvant groups, were used to predict each antibody response feature on the three timepoints. The models also allowed us to rank the abovementioned innate immune variables that were found to be predictive of each antibody feature on each of these timepoints (see **Supplementary Table 12** for detailed modeling results and feature ranking).

**Figure 4:**
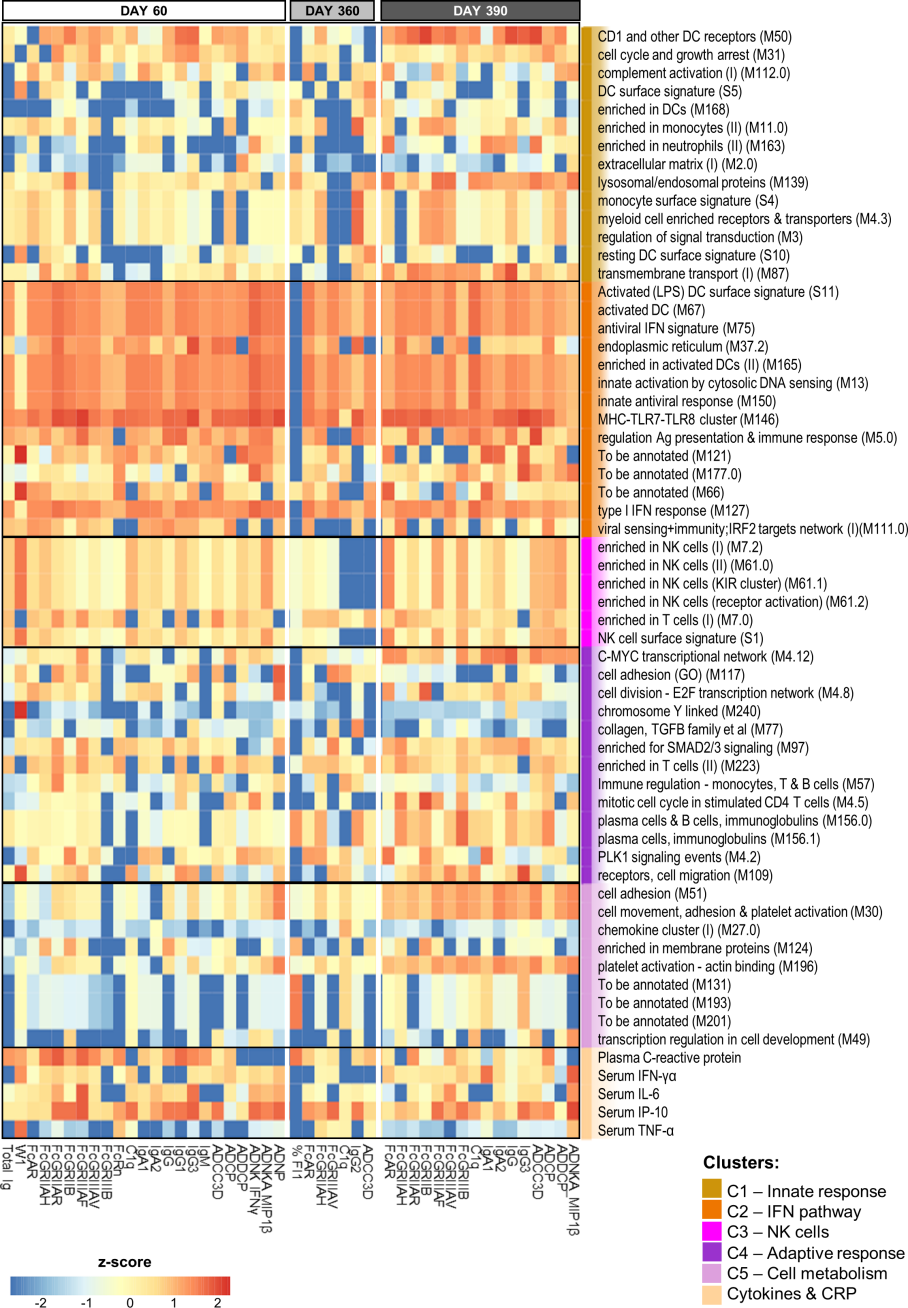
BTM-based predictive response signature persists over time. Heatmap of the union of the top 10 selected innate response features (including log fold-change D31/D30 serum cytokine and C-reactive protein [CRP] levels and blood transcriptional model [BTM] enrichments) that have the highest rank/power to predict an antibody response feature on days 60, 360 and 390 across the adjuvant groups. Rankings derived by LASSO and Random Forest were averaged and standardized by antibody response feature. Tile colors correspond to the scaled ranks of the innate response, where red/blue correspond to higher/lower predictive power. Row annotations represent the BTMs and functional BTM clusters assigned by K-means clustering using the analysis pipeline and cluster definitions based on a previously described method (27).

We then plotted the normalized ranks of the top 10 (C1-C5 annotated) innate variables (rows) against each predictable antibody feature (columns) by timepoint (**Figure 4**; accuracy >50%). Prediction powers of the (mostly shared) innate variables were similar between D60 and D390, but lower at D360, when fewer predictable antibody features were found relative to D60 or D390 (i.e., 7 vs 23 or 16 respectively). Indeed, at D60, the predicted features comprised the total Ig titer, six class/isotype-specific Ig titers, W1, C1q-binding antibodies, eight Fc-binding functions, and six AD functions. At D360, this number had waned to three Fc-related features, two timepoint-specific features (namely IgG_2_ and %FI_1_), C1q-binding antibodies, and ADCC3D. A likely explanation of this reduction in functions was that the assay sensitivities did not allow detecting the low values of most of the AD features and titers that occurred at this late timepoint. The number of features expanded again on D390, to seven Fc-mediated features, four AD features, C1q-binding antibodies, and four subtype/class-specific Ig titers.

Interestingly, the parameters that proved to be highly predictive (i.e., predicting >3 antibody features at each timepoint) comprised, besides CRP and IP-10 responses, predominantly IFN pathway-related (C2) BTMs. These IFN-related modules were involved in regulating activated DCs (S11, M67, and M165), antigen presentation (M5.0), antiviral/IFN-related responses (M75, M150, and M127), cytosolic DNA sensing (M13), MHC TLR7/8 functions (M146), and the endoplasmic reticulum (ER; M37.2). Avidity parameters were not predictable by this signature, but were predictable by a number of different BTMs. Indeed, W1 at D60 was predicted by NK-related (C3) responses and CRP, IFN-γ and TNF-α protein responses, and %FI1 at D360 was predicted by plasma-related (M156.0 and M156.1) and CRP responses (as well as by three non-annotated BTMs). Of note, for W1, the explained variances of the classification-based prediction models were adequate on D60 [i.e., ~60–70% for BTMs (**Supplementary Table 4**) and ~70% for genes (**Supplementary Table 5**)], but only ~50% on the other timepoints. Finally, the data pointed to the presence of a distinct predictive signature after the antigenic challenge. Indeed, several predictors of the D390 response were timepoint-specific, such as the modules related to either platelet activation (M196, M30, and M51) or plasma cells (M156.0 and M156.1).

**Figure 5:**
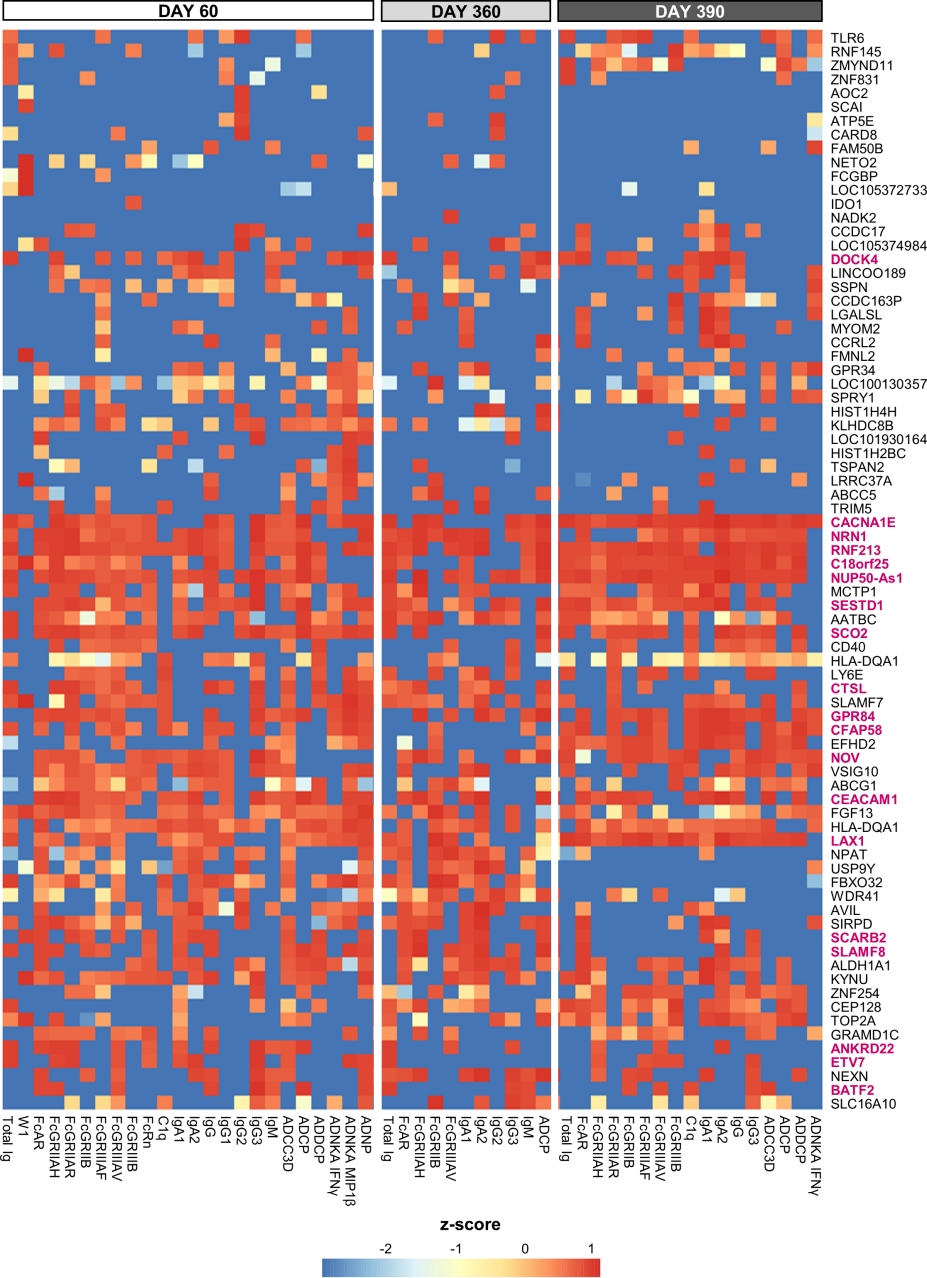
Gene-based signature consistently predicts antibody features. Heatmap of the union of the top 5 selected innate response features (including log fold-change D31/D30 serum cytokine and C-reactive protein [CRP] levels and gene expression values) that have the highest rank or predictive power to predict each antibody response feature on each day (day 60, 360 and 390) across the adjuvant groups (see **Supplementary Figure 8** for the top 10 selected features). Rankings derived by LASSO and Random Forest were averaged and standardized by antibody response feature. Tile colors correspond to the scaled ranks of the innate response, where red/blue corresponds to higher/lower predictive power (rather than to the direction of gene regulation). Row annotations represent the gene names, with the 19 cluster-representative genes included in **Table 2** highlighted in pink font.

**Figure 6:**
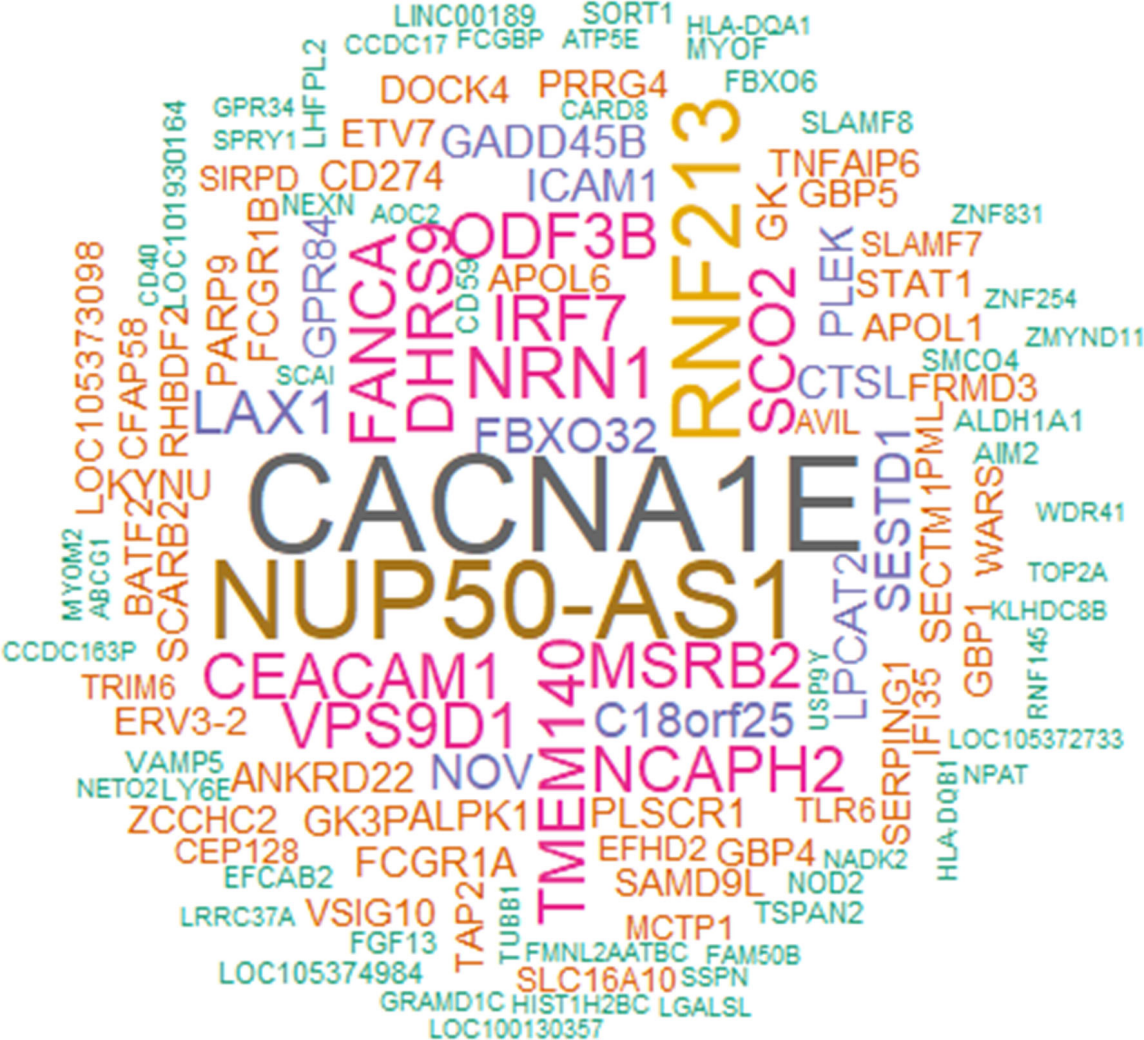
Genes predictive of the longitudinal antibody response. The word cloud represents the union of the top 5 genes able to predict the longitudinal antibody response (see **Supplementary Figure 10** for the word cloud related to the top 10 genes). Data were derived from all participants across all adjuvant groups and all timepoints among days 60, 360 and 390. The font size is proportional to the total number of predicted antibody response variables on a timepoint, with the specific timepoints detailed by gene in **Supplementary Table 13** (first tab).

From a similar analysis of the predictive D31/D30 gene expression, we derived a union of the top 5 or top 10 innate response features (**Figure 5** or **Supplementary Figure 9**, respectively; see legends for details). In these unions of genes, each (IQR-selected) gene represented a set of highly correlated genes (Pearson correlation >80%), as identified for each predictable antibody feature; see **Supplementary Table 1** for the complete ranked list of the predictor genes and associated details. The union of the top 5 genes confirmed the kinetic pattern in antibody predictability seen in the BTMs (**Figure 4**), by showing 24, 11 and 17 predictable antibody responses at D60, D360 and D390, respectively. We then considered all genes per cluster of gene representatives (**Table 2**; see **Supplementary Table 13** for the full list of all genes, cluster-representative genes, and the number of antibody features predicted by each cluster). The gene signature that emerged consisted of the shared predictors of the longitudinal antibody response, with CACNA1E, NUP50-AS1 and RNF213 as highest ranking genes (**Figure 6**; **Supplementary Figure 10** for the top5 and top 10 gene data, respectively). The data confirmed that the top-ranking predictors were mostly genes involved in the IFN-signaling pathway, immune regulation, and transcription factor motif enrichment (**Supplementary Tables 9**, **10** for top 5/top 10 genes). Finally, we compared these gene-sets with the 300 DEGs previously identified as being associated with the peak titers (27). This analysis showed that the majority (>61% at D60, >58% at D360, and >51% at D390) of the currently identified (top 5) antibody titer/quality-predictive genes were among these previously identified titer-associated DEGs.

**Table 2:** Gene clusters most predictive of antibody response features across days 60, 360 and 390.

Gene cluster predictive of ≥10 antibody response features (listed in **Figure 4**) on a given timepoint^1^	Number of predicted antibody features for which the identified gene cluster ranked among top 10 predictors
***CACNA1E***	35
***RNF213***	34
***NUP50-AS1***	29
*DHRS9*, ***CEACAM1*** *, MSRB2, TMEM140*	23
*NCAPH2, IRF7, FANCA, ODF3B, VPS9D1*, ***SCO2***	21
*LPCAT2*, ***GPR84*** *, ICAM1, PLEK, GADD45B*	16
***NRN1***	16
***LAX1***	15
***C18orf25***	14
***NOV***	14
***SESTD1***	13
*FBXO6, NOD2, LHFPL2*, ***SLAMF8*** *, MYOF, SORT1*	13
*PLSCR1, FCGR1A, ALPK1, PARP9*, ***ANKRD22*** *, FCGR1B, GK, CD274, SECTM1, GK3P*	12
*GBP4, WARS, PML*, ***BATF2*** *, SAMD9L, GBP5, TAP2, STAT1, FRMD3, RHBDF2, APOL1, SERPING1, GBP1, IFI35*	12
*TNFAIP6, LOC105373098, ERV3-2, ZCCHC2*, ***DOCK4***	12
***CFAP58***	11
***CTSL***	11
***ETV7*** *, APOL6, PRRG4*	11
***SCARB2***	10

^1^Cluster-representative genes (see Methods on hierarchical clustering of genes/blood transcriptional modules) are shown in bold underlined font.

Thus, with the possible exception of avidity-related variables, the shared innate signature – consisting of D31/D30 IFN-pathway-related gene expression and secreted IP-10 and CRP levels – was predictive of both the quantitative and the qualitative antibody response features reflecting the impacts of the full vaccination regimen, a year-long antibody waning, and the antigenic challenge.

### Groups of adjuvants can be stratified by the innate immune features they promote

**Figure 7:**
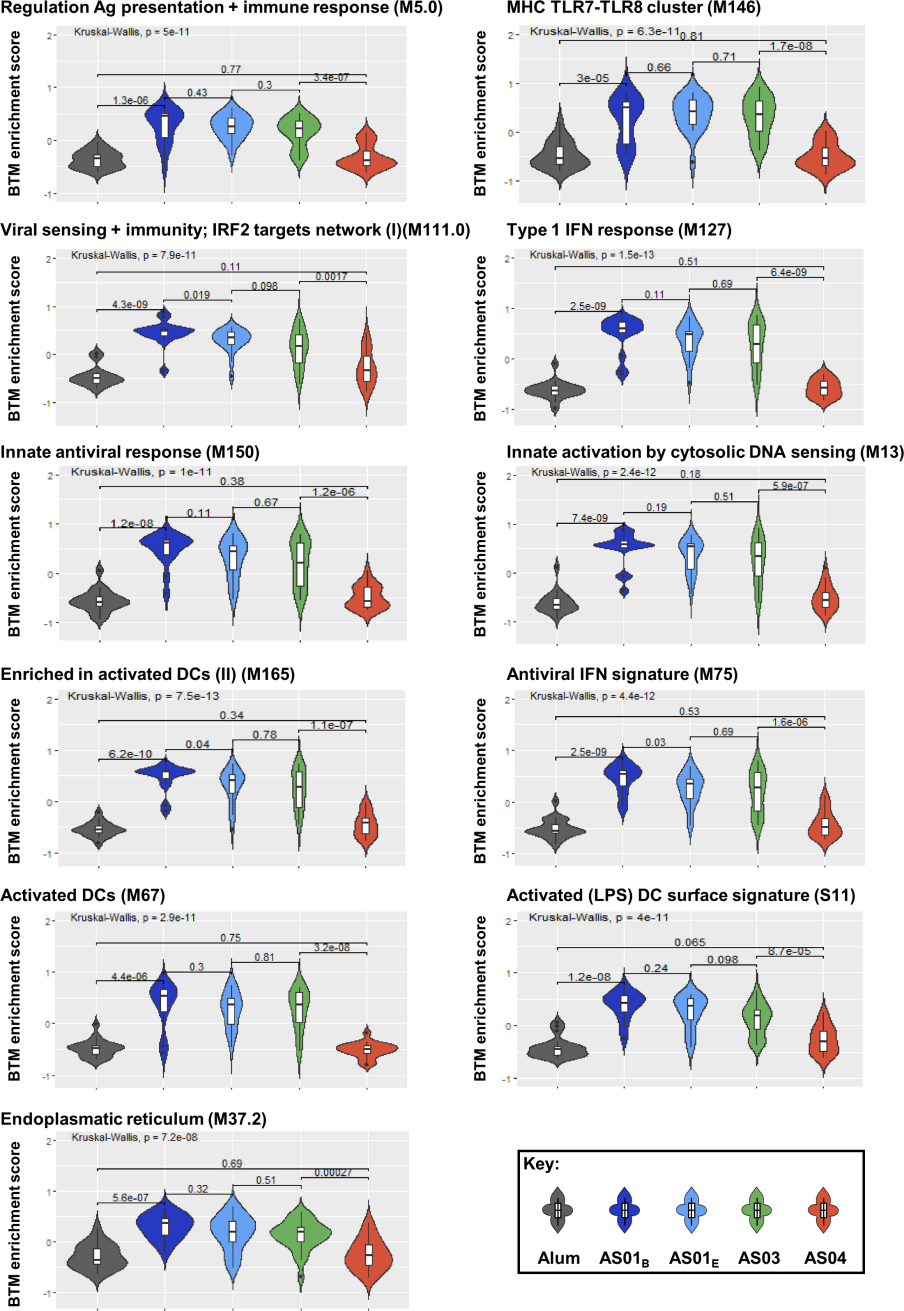
Innate responses predicting adjuvant groups are mainly induced by AS01 and AS03 and center on the IFN pathway. Violin plots present the distribution of the enrichment scores by adjuvant group for the predefined (30) blood transcriptional models (BTMs; D31/D30 contrast, with names indicated above each plot) which were identified as discriminators of the groups of adjuvants (see **Table 3**; **Supplementary Table 3**). Numerical values within the plots represent the Benjamini-Hochberg-adjusted p-values, and are indicated both overall (top horizontal bar) and by individual comparison obtained by Kruskal-Wallis tests (lower horizontal bars).

Given the strong associations between the innate/IFN pathway-related modules and the functional antibody response seen thus far, and considering that such responses are the hallmark of the immunity promoted by the AS01_B_/AS01_E_/AS03 group of adjuvants (26, 27), we then investigated to what extent these modules can be used as markers for the adjuvant group administered in a vaccination. Using an extended version of the previously applied (21) pipeline, we compared the two main groups of adjuvants based on the systems serology features they induced. This pipeline (**Supplementary Figure 2**) was applied to the full innate data set and then the classifying features were identified. We found that the 11 BTMs that were selected to discriminate between the two main groups were all involved in the IFN pathway (**Table 3**; **Supplementary Table 3**). Interestingly, the median enrichment scores found for each of these BTMs were significantly higher for the AS01_B_/AS01_E_/AS03 group of adjuvants (**Figure 7**).

**Table 3:** LASSO-selected BTMs in comparison data from AS01/AS03 vs AS04/Alum.

BTM	% AS01/AS03 vs AS04/Alum^1^
Regulation of antigen presentation and immune response (M5.0)	100.00
MHC-TLR7-TLR8 cluster (M146)	100.00
Viral sensing & immunity; IRF2 targets network (I) (M111.0)	99.00
Type I IFN response (M127)	94.33
Innate antiviral response (M150)	94.33
Innate activation by cytosolic DNA sensing (M13)	94.33
Enriched in activated DCs (II) (M165)	94.33
Antiviral IFN signature (M75)	94.33
Activated DCs (M67)	94.33
Activated (LPS) DC surface signature (S11)	94.33
Endoplasmic reticulum (M37.2)	81.00

**^1^** Frequencies refer to the number of times a feature was selected by LASSO (adjusted for highly correlated features) as belonging to the AS01/AS03 adjuvant group; see **Supplementary Table 3** for accuracy details of the model. BTM, Blood transcriptional module, MHC, major histocompatibility complex. TLR, Toll-like receptor. IFN, interferon. DC, dendritic cell. LPS, lipopolysaccharide. Data were generated using an adjusted version of the pipeline described in ref (21). Model accuracies were 0.908 overall and 0.551 for the null/intercept model (p-value = 1.22 E-14).

Along with the data in **Figure 4**, we conclude that a shared innate immune signal can predict the comprehensive set of antibody response features in individuals administered adjuvanted HBsAg vaccines after a short or a prolonged interval post second vaccination. This innate signal is mainly characterized by IFN pathway-related functions, which constitutes the marker for responses promoted by AS01 and AS03. Once confirmed, this data could serve as a basis for future models designed to predict from vaccinee’s immune response, whether this individual has received a vaccine containing a member of this main group of adjuvants.

## Discussion

Recent years have seen an increased focus on identifying predictive markers of vaccine responses, including humoral response markers and even potentially predictive efficacy markers (31–33). The markers emerging from these studies, such as cytokine expression at the gene or protein level, were mostly predictive of the antibody quantity/titer measured a few weeks after vaccination. While vaccine development certainly benefits from these quantitative predictors of the humoral response, extending the predictions to the response persistence or antibody functionality/quality would increase the interest of such research, as the induction of functional antibodies is an essential attribute for many vaccines against critical infectious or non-infectious diseases (3–8). Previously, a core innate signature emerging after the second but not the first vaccination with AS01- or AS03-adjuvanted HBsAg vaccines was shown to be associated with the antibody titers measured one month post second vaccination (26, 27). Here we extended these analyses to prediction, i.e., using the innate response as a predictor for the adaptive response rather than as an association. We identified a gene signature that was able to predict not only the peak titer but also the persistence of the humoral response in its globality after a year, and, interestingly, also after a subsequent antigenic challenge. This study is thus unique in showing that not only the quantitative but also the qualitative features of the antibody response are predictable by a specific transcriptional signature that is composed of D31/D30 DEGs centering on IFN response-related functions. Finally, our data highlighted distinct abilities of the adjuvants in inducing predictive signatures, by separating AS01 and AS03 from AS04 and Alum, aligned with the preceding reports (21, 26–29).

The antibody response predictors identified for AS01 and AS03 consisted of enriched BTMs related to antigen presentation pathways, including MHC-related, TLR7/8-related, and activated DC-related gene modules, as well as modules relating to the IFN pathway. The latter included both IFN type I responses (see **Figure 4**) and ER-related modules. Overall, these BTMs reflected the DC-activated functions operating in the progression of the antiviral response, i.e., from viral recognition and proinflammatory cytokine/type I IFN production, to the generation of adaptive immunity. Indeed, in immature DCs, MHC I or II molecules are loaded with antigenic peptides in the ER or ER-connected endosomal compartments, respectively. Upon activation by inflammatory stimuli and maturation, these cells will express antigen-loaded MHC II at their membranes and migrate to lymphoid organs, where they provide the signals initiating an effective adaptive response (34). Interestingly, the predictive capacity of these antigen-presentation modules was not limited to the antibody response measured 1 month after the last vaccination, but was similarly predictive of the persistent antibody response, regardless of whether this concerned the titers or the qualitative features. Furthermore, the emergence of predictive plasma cell-related functions after the challenge may extend previous findings for the AS01-adjuvanted malaria vaccine (35). That study demonstrated correlations between the B-cell activation-related gene expression and plasmablast responses post-booster, as well as between the modules related to antigen presentation, DC activation and antiviral/type I IFN responses, and the malaria antigen-specific antibody titers. While NK-related modules were negatively associated with the antibody titers at 3 weeks post-malaria challenge (35), we observed here that NK modules enabled the (low power) prediction of certain long-term persisting functional antibody features (ADCC3D, IgG2 and C1q), again highlighting the importance of performing longitudinal assessments of the antibody functionality.

For AS01/AS03, we observed that the predictors identified 1 month after the full vaccination schedule, 1 year later, or 1-month post-challenge were overall of a comparable nature. Interestingly, the set of modules found to be predictive of most of the qualitative or quantitative antibody features was similar to the predictors identified for the two 1-month timepoints. Although unverifiable due to the lack of an adjuvanted challenge vaccination, this suggests that an adjuvant would be less needed for the third dose of these vaccines, as proposed previously (21). This may be explained by memory B-cell programming upon an antigenic exposure in the presence of an effective adjuvant, which can encode the response features also after a non-adjuvanted boost or anamnestic response. This would align with non-human primate data showing that prime-boost regimens of vector-based vaccines induced phenotypic changes in innate myeloid and NK cells (36–39), and may possibly also implicate the presence of innate imprinting. Indeed, innate memory elicited by these two adjuvant systems can persist for at least 6 months, and involves changes in transcriptional modules (notably those linked to the IFN response) with a key role for monocytes and DCs [unpublished data and (40)], hinting at a readiness of innate cells to respond to an antigenic challenge even in the absence of an adjuvant. Furthermore, we noted that the largely overlapping signatures of predictable antibody features for both 1-month timepoints differed from the signature for the 1-year persisting antibody response which contained fewer predictive modules. It is unclear whether this reflected a distinct decrease in the overall antibody response to undetectable levels, or a qualitative decay of the antibody response. While the mechanisms underlying the regulation of Fc-mediated features remain to be explored, for avidity-related parameters, this matter could be investigated by evaluating the activation of pathways regulating processes such as oxidative phosphorylation and SREBP1 (41, 42), which underpin B-cell activation and affinity maturation in long-lived plasma cells and lead to durable high-avidity antibodies. Altogether, the signature identified here shows that a specific signature obtained after two doses of AS01/AS03-containing vaccines can help mounting a robust functional antibody response, also after a non-adjuvanted antigenic stimulus.

Confirming the dichotomous nature of the responses promoted by these groups of adjuvants (21, 26, 27), we found that the shared signatures of antibody quantity/functionality were mostly predictive of AS01/AS03. The low number of predictors in AS04/Alum-induced responses may be related to our timepoint selection, which did not accommodate the delayed response kinetics seen for these two adjuvants (27). Alternatively, it may be related to the analytical thresholds for innate response detection, at least in blood, though responses might be detectable in the lymph nodes. Even for AS01 and AS03, the predictive markers could only be identified for the D31/D30 ratio, after completion of the two-dose immunization regimen. Indeed, the predictability of these markers was in fact much lower when analyzing the D31 data relative to the D0 baseline (data not shown), suggesting that the first immunization had introduced changes which were not apparent when directly comparing D31 and D0. While a common transcriptional signature is shared by many viral, protein and anti-polysaccharide vaccines, its response kinetics can vary considerably between vaccines and adjuvants (33, 43) — case in point being the distinct kinetics of gene expression promoted by AS04 or Alum vs AS01 or AS03 (27). Adjusting for the time of peak gene expression may circumvent such kinetic differences. This approach enabled Hagan et al. to identify Ig responses and plasma cell-related gene expression (M156.1) at D7 as common antibody titer predictors, but at timepoints that could vary by 1 or 2 weeks across the investigated vaccines (33). Interestingly, besides also identifying M156.1 as predictive marker in our data, we found that the associated plasma-cell-related module M156.0 was predictive of not only the antibody titer, but also of antibody functionality. These two modules as well as the platelet-activation BTMs (M196, M30, M51) predicted the antibody response mostly after the antigenic challenge. However, the predictive modules that were consistently detected in the current participants predominantly centered on activated DC/IFN-related responses, rather than on the plasma responses or platelet activation seen in the reference study (33). This suggests a role for pre-sensitization of the innate immune system to PAMPs in priming the B-cell response to vaccination (44). Alternatively, the differences could be explained by the choice of study vaccines [a single antigen here, vs 13 vaccines excluding HBsAg in the reference study (33)] and/or by our approach of considering both antibody functionality and titers.

Further research is needed into the influence of CRP and IL-6 responses after the second dose. These protein responses promoted by AS01 and AS03 were found to be not predictive of the comprehensive antibody response, in contrast to the IP-10 response, while all three parameters were previously shown to correlate with the D60 antibody titer [for AS01/AS03 (26)] as well as with systemic reactogenicity symptoms [for AS01 (45)]. Whether these observations strictly reflected a statistical association without any role in antibody maturation/titers, or the inflammation underlying the reactogenicity symptoms, could be explored in animal models, by minimizing such protein responses without influencing IFN pathway induction.

This study has several potential limitations. First, as the participants were antigen-naïve, the generalizability of our findings for primed populations remains to be investigated. Indeed, the presence of pre-existing antibody titers can diminish the induction of IFN and plasma cell signatures (44), but the possible impact of the quality of a baseline antibody response on innate immunity is unknown—as is the applicability of our data for other adjuvants, antigens, or vaccine platforms (such as mRNA or vector-based vaccines), or for more durable (>1 year persisting) antibody responses. These aspects warrant further exploration in future studies. Besides the vaccine-specific response kinetics, as mentioned, a host of other factors can confound cross-vaccine and/or cross-population comparisons of biomarkers for antibody functions, including for example inter-individual variability in microbiomes (46, 47). Finally, while the robustness of our approach was demonstrated using different analytical tools in separate analyses, the obtained model accuracies (see Methods) will need to be verified using larger datasets. Once confirmed to be generalizable in broader context, the identified signature can pave the way for the development of a simple assay [‘vaccine chip’ (23, 33)] to measure the expression of a predefined minimal selection of shared predictor genes or BTMs, in order to predict specific functional antibody features. Such application can support the development of personalized medicine/vaccination strategies [as defined in a different context (48)], in contrast to the contributions to precision vaccination offered by the previous reports (26, 27).

Altogether, our data represent an important link in the chain of successive reports capturing a broad array of immune features involved in the progression from innate to adaptive responses. As this data was generated for five clinically relevant adjuvants with a well-characterized antigen and in a single population, it has comprehensively elucidated the mode of action of these adjuvants in humans (21, 26–29). Our study is also unique in that it predicts for the first time, to the best of our knowledge, both the antibody titers and quality rather than strictly the titers for adjuvanted vaccines. To conclude, a shared innate immune signal characterized by the IFN pathway-related response, a marker of the response promoted by AS01 or AS03, can predict quantitative and qualitative antibody features persisting after completion of a vaccination regime.

## Data availability statement

GSK makes available anonymized individual participant data and associated documents from interventional clinical studies which evaluate medicines, upon approval of proposals submitted to www.clinicalstudydatarequest.com. To access data for other types of GSK-sponsored research, for study documents without patient-level data, and for clinical studies not listed, please submit an inquiry via the website (GSK study identifier: 112115/ClinicalTrials.gov identifier: NCT00805389). The computer code used for the presented analysis is available at Zenodo (https://zenodo.org/record/12092338). Other datasets presented in this study can be found in online repositories. The names of the repository/repositories and accession number(s) can be found in the article/**Supplementary Material**.

## Ethics statement

The studies involving humans were approved by all institutional Ethics Committees and conducted in accordance with the Helsinki Declaration and Good Clinical Practice guidelines. The studies were conducted in accordance with the local legislation and institutional requirements. The participants provided their written informed consent to participate in this study.

## Author contributions

ST: Conceptualization, Formal analysis, Investigation, Software, Validation, Visualization, Writing – original draft, Writing – review & editing. VB: Conceptualization, Investigation, Project administration, Supervision, Writing – original draft, Writing – review & editing. AE: Data curation, Methodology, Project administration, Writing – review & editing. YS: Conceptualization, Methodology, Supervision, Writing – review & editing. WB: Conceptualization, Supervision, Writing – review & editing.

## Author disclaimer

Fendrix, Engerix-B, AS01, and AS03 are trademarks owned by or licensed to GSK.

## Supplementary material

Supplementary Table 1 Gene cluster representatives and gene ranking for predictive modeling of antibody features.

Supplementary Table 2 Wilcoxon rank-sum test-based detection of significant differences between adjuvant groups.

Supplementary Table 3 Final adjuvant classification, feature selection and accuracies for the integrated data-set.

Supplementary Table 4 R^2^ scores of the predictive modeling based on blood transcriptional models.

Supplementary Table 5 R^2^ scores of the gene-based predictive modeling.

Supplementary Table 6 W1 statistics of the predictive modeling based on blood transcriptional models.

Supplementary Table 7 W1 statistics of the gene-based predictive modeling.

Supplementary Table 8 Correlations between rankings obtained by LASSO-based vs Random Forest-based methods.

Supplementary Table 9 GO enrichments for the union of top 5 genes predictive of timed antibody response features.

Supplementary Table 10 GO enrichments for the union of top 10 genes predictive of timed antibody response features.

Supplementary Table 11 Blood transcriptional model identification and enrichment scores.

Supplementary Table 12 Blood transcriptional model ranking based on the predictive modeling.

Supplementary Table 13 All genes, cluster representative genes, and numbers of antibody features predicted by each cluster.

## References

[1] Singh G Abbad A Tcheou J Mendu DR Firpo-Betancourt A Gleason C. Binding and avidity signatures of polyclonal sera from individuals with different exposure histories to severe acute respiratory syndrome coronavirus 2 infection, vaccination, and omicron breakthrough infections. J Infect Dis. (2023) 228:564–75. doi: 10.1093/infdis/jiad116 PMC10469125 37104046

[2] Chung AW Kumar MP Arnold KB Yu WH Schoen MK Dunphy LJ. Dissecting polyclonal vaccine-induced humoral immunity against HIV using systems serology. Cell. (2015) 163:988–98. doi: 10.1016/j.cell.2015.10.027 PMC5490491 26544943

[3] Boudreau CM Burke JS Yousif AS Sangesland M Jastrzebski S Verschoor C. Antibody-mediated NK cell activation as a correlate of immunity against influenza infection. Nat Commun. (2023) 14:5170. doi: 10.1038/s41467-023-40699-8 37620306 PMC10449820

[4] Bartsch YC Cizmeci D Kang J Zohar T Periasamy S Mehta N. Antibody effector functions are associated with protection from respiratory syncytial virus. Cell. (2022) 185:4873–86. doi: 10.1016/j.cell.2022.11.012 36513064

[5] Lo Russo G Moro M Sommariva M Cancila V Boeri M Centonze G. Antibody-fc/fcR interaction on macrophages as a mechanism for hyperprogressive disease in non-small cell lung cancer subsequent to PD-1/PD-L1 blockade. Clin Cancer Res. (2019) 25:989–99. doi: 10.1158/1078-0432.CCR-18-1390 30206165

[6] Goldblatt D Alter G Crotty S Plotkin SA. Correlates of protection against SARS-CoV-2 infection and COVID-19 disease. Immunol Rev. (2022) 310:6–26. doi: 10.1111/imr.13091 35661178 PMC9348242

[7] Musolino A Gradishar WJ Rugo HS Nordstrom JL Rock EP Arnaldez F. Role of Fcγ receptors in HER2-targeted breast cancer therapy. J Immunother Cancer. (2022) 10:e003171. doi: 10.1136/jitc-2021-003171 34992090 PMC8739678

[8] Suscovich TJ Fallon JK Das J Demas AR Crain J Linde CH. Mapping functional humoral correlates of protection against malaria challenge following RTS,S/AS01 vaccination. Sci Transl Med. (2020) 12:eabb4757. doi: 10.1126/scitranslmed.abb4757 32718991

[9] Eisen HN. Affinity enhancement of antibodies: how low-affinity antibodies produced early in immune responses are followed by high-affinity antibodies later and in memory B-cell responses. Cancer Immunol Res. (2014) 2:381–92. doi: 10.1158/2326-6066.CIR-14-0029 24795350

[10] Benner SE Patel EU Laeyendecker O Pekosz A Littlefield K Eby Y. SARS-coV-2 antibody avidity responses in COVID-19 patients and convalescent plasma donors. J Infect Dis. (2020) 222:1974–84. doi: 10.1093/infdis/jiaa581 PMC7499592 32910175

[11] Bauer G. The potential significance of high avidity immunoglobulin G (IgG) for protective immunity towards SARS-CoV-2. Int J Infect Dis. (2021) 106:61–4. doi: 10.1016/j.ijid.2021.01.061 PMC7944804 33713819

[12] Pegu P Vaccari M Gordon S Keele BF Doster M Guan Y. Antibodies with high avidity to the gp120 envelope protein in protection from simian immunodeficiency virus SIVmac251 acquisition in an immunization regimen that mimics the RV-144 Thai trial. J Virol. (2013) 87:1708–19. doi: 10.1128/JVI.02544-12 PMC3554145 23175374

[13] Junker AK Tilley P. Varicella-zoster virus antibody avidity and IgG-subclass patterns in children with recurrent chickenpox. J Med Virol. (1994) 43:119–24. doi: 10.1002/jmv.1890430204 8083659

[14] Delgado MF Coviello S Monsalvo AC Melendi GA Hernandez JZ Batalle JP. Lack of antibody affinity maturation due to poor Toll-like receptor stimulation leads to enhanced respiratory syncytial virus disease. Nat Med. (2009) 15:34–41. doi: 10.1038/nm.1894 19079256 PMC2987729

[15] Boppana SB Britt WJ. Antiviral antibody responses and intrauterine transmission after primary maternal cytomegalovirus infection. J Infect Dis. (1995) 171:1115–21. doi: 10.1093/infdis/171.5.1115 7751685

[16] Pulendran B Arunachalam S O’Hagan DT. Emerging concepts in the science of vaccine adjuvants. Nat Rev Drug Discov. (2021) 20:454–75. doi: 10.1038/s41573-021-00163-y PMC8023785 33824489

[17] Weinberg A Schmid DS Leung J Johnson MJ Miao C Levin MJ. Predictors of five-year persistence of antibody responses to zoster vaccines. J Infect Dis. (2023) 228:1367–74. doi: 10.1093/infdis/jiad132 PMC10640777 37141390

[18] Francica JR Zak DE Linde C Siena E Johnson C Juraska M. Innate transcriptional effects by adjuvants on the magnitude, quality, and durability of HIV envelope responses in NHPs. Blood Adv. (2017) 1:2329–42. doi: 10.1182/bloodadvances.2017011411 PMC5729628 29296883

[19] Thompson EA Ols S Miura K Rausch K Narum DL Spangberg M. TLR-adjuvanted nanoparticle vaccines differentially influence the quality and longevity of responses to malaria antigen Pfs25. JCI Insight. (2018) 3:e120692. doi: 10.1172/jci.insight.120692 29769448 PMC6012510

[20] Kasturi SP Kozlowski PA Nakaya HI Burger MC Russo P Pham M. Adjuvanting a simian immunodeficiency virus vaccine with toll-like receptor ligands encapsulated in nanoparticles induces persistent antibody responses and enhanced protection in TRIM5alpha restrictive macaques. J Virol. (2017) 91(4):e01844-16. doi: 10.1128/JVI.01844-16 27928002 PMC5286877

[21] Loos C Coccia M Didierlaurent AM Essaghir A Fallon JK Lauffenburger D. Systems serology-based comparison of antibody effector functions induced by adjuvanted vaccines to guide vaccine design. NPJ Vaccines. (2023) 8:34. doi: 10.1038/s41541-023-00613-1 36890168 PMC9992919

[22] Arunachalam PS Walls AC Golden N Atyeo C Fischinger S Li C. Adjuvanting a subunit COVID-19 vaccine to induce protective immunity. Nature. (2021) 594:253–8. doi: 10.1038/s41586-021-03530-2 33873199

[23] Pulendran B Li S Nakaya HI. Systems vaccinology. Immunity. (2010) 33:516–29. doi: 10.1016/j.immuni.2010.10.006 PMC3001343 21029962

[24] Pulendran B. Systems vaccinology: probing humanity’s diverse immune systems with vaccines. Proc Natl Acad Sci USA. (2014) 111:12300–6. doi: 10.1073/pnas.1400476111 PMC4151766 25136102

[25] Nakaya HI Hagan T Duraisingham SS Lee EK Kwissa M Rouphael N. Systems analysis of immunity to influenza vaccination across multiple years and in diverse populations reveals shared molecular signatures. Immunity. (2015) 43:1186–98. doi: 10.1016/j.immuni.2015.11.012 PMC4859820 26682988

[26] Burny W Callegaro A Bechtold V Clement F Delhaye S Fissette L. Different adjuvants induce common innate pathways that are associated with enhanced adaptive responses against a model antigen in humans. Front Immunol. (2017) 8:943. doi: 10.3389/fimmu.2017.00943 28855902 PMC5557780

[27] De Mot L Bechtold V Bol V Callegaro A Coccia M Essaghir A. Transcriptional profiles of adjuvanted hepatitis B vaccines display variable interindividual homogeneity but a shared core signature. Sci Transl Med. (2020) 12:eaay8618. doi: 10.1126/scitranslmed.aay8618 33177181

[28] Leroux-Roels G Marchant A Levy J Van Damme P Schwarz TF Horsmans Y. Impact of adjuvants on CD4(+) T cell and B cell responses to a protein antigen vaccine: Results from a phase II, randomized, multicenter trial. Clin Immunol. (2016) 169:16–27. doi: 10.1016/j.clim.2016.05.007 27236001

[29] Budroni S Buricchi F Cavallone A Bourguignon P Caubet M Dewar V. Antibody avidity, persistence, and response to antigen recall: comparison of vaccine adjuvants. NPJ Vaccines. (2021) 6:78. doi: 10.1038/s41541-021-00337-0 34021167 PMC8140094

[30] Li S Rouphael N Duraisingham S Romero-Steiner S Presnell S Davis C. Molecular signatures of antibody responses derived from a systems biology study of five human vaccines. Nat Immunol. (2014) 15:195–204. doi: 10.1038/ni.2789 24336226 PMC3946932

[31] Chaudhury S Duncan EH Atre T Storme CK Beck K Kaba SA. Identification of immune signatures of novel adjuvant formulations using machine learning. Sci Rep. (2018) 8:17508. doi: 10.1038/s41598-018-35452-x 30504893 PMC6269591

[32] Van Tilbeurgh M Lemdani K Beignon AS Chapon C Tchitchek N Cheraitia L. Predictive markers of immunogenicity and efficacy for human vaccines. Vaccines (Basel). (2021) 9(6):579. doi: 10.3390/vaccines9060579 34205932 PMC8226531

[33] Hagan T Gerritsen B Tomalin LE Fourati S Mule MP Chawla DG. Transcriptional atlas of the human immune response to 13 vaccines reveals a common predictor of vaccine-induced antibody responses. Nat Immunol. (2022) 23:1788–98. doi: 10.1038/s41590-022-01328-6 PMC9869360 36316475

[34] Banchereau J Briere F Caux C Davoust J Lebecque S Liu YJ. Immunobiology of dendritic cells. Annu Rev Immunol. (2000) 18:767–811. doi: 10.1146/annurev.immunol.18.1.767 10837075

[35] Kazmin D Nakaya HI Lee EK Johnson MJ van der Most RG van den Berg RA. Systems analysis of protective immune responses to RTS,S malaria vaccination in humans. Proc Natl Acad Sci USA. (2017) 114:2425–30. doi: 10.1073/pnas.1621489114 PMC5338562 28193898

[36] Palgen JL Tchitchek N Huot N Elhmouzi-Younes J Lefebvre C Rosenbaum P. NK cell immune responses differ after prime and boost vaccination. J Leukoc Biol. (2019) 105:1055–73. doi: 10.1002/JLB.4A1018-391RR 30794328

[37] Palgen JL Tchitchek N Elhmouzi-Younes J Delandre S Namet I Rosenbaum P. Prime and boost vaccination elicit a distinct innate myeloid cell immune response. Sci Rep. (2018) 8:3087. doi: 10.1038/s41598-018-21222-2 29449630 PMC5814452

[38] Palgen JL Tchitchek N Rodriguez-Pozo A Jouhault Q Abdelhouahab H Dereuddre-Bosquet N. Innate and secondary humoral responses are improved by increasing the time between MVA vaccine immunizations. NPJ Vaccines. (2020) 5:24. doi: 10.1038/s41541-020-0175-8 32218996 PMC7081268

[39] Rosenbaum P Tchitchek N Joly C Stimmer L Hocini H Dereuddre-Bosquet N. Molecular and cellular dynamics in the skin, the lymph nodes, and the blood of the immune response to intradermal injection of modified vaccinia ankara vaccine. Front Immunol. (2018) 9:870. doi: 10.3389/fimmu.2018.00870 29922280 PMC5996922

[40] Wimmers F Donato M Kuo A Ashuach T Gupta S Li C. The single-cell epigenomic and transcriptional landscape of immunity to influenza vaccination. Cell. (2021) 184:3915–35.e21. doi: 10.1016/j.cell.2021.05.039 34174187 PMC8316438

[41] Luo W Adamska JZ Li C Verma R Liu Q Hagan T. SREBP signaling is essential for effective B cell responses. Nat Immunol. (2023) 24:337–48. doi: 10.1038/s41590-022-01376-y PMC10928801 36577930

[42] Chen D Wang Y Manakkat Vijay GK Fu S Nash CW Xu D. Coupled analysis of transcriptome and BCR mutations reveals role of OXPHOS in affinity maturation. Nat Immunol. (2021) 22:904–13. doi: 10.1038/s41590-021-00936-y 34031613

[43] Weiner J Lewis DJM Maertzdorf J Mollenkopf HJ Bodinham C Pizzoferro K. Characterization of potential biomarkers of reactogenicity of licensed antiviral vaccines: randomized controlled clinical trials conducted by the BIOVACSAFE consortium. Sci Rep. (2019) 9:20362. doi: 10.1038/s41598-019-56994-8 31889148 PMC6937244

[44] Fourati S Tomalin LE Mule MP Chawla DG Gerritsen B Rychkov D. Pan-vaccine analysis reveals innate immune endotypes predictive of antibody responses to vaccination. Nat Immunol. (2022) 23:1777–87. doi: 10.1038/s41590-022-01329-5 PMC9747610 36316476

[45] Burny W Marchant A Hervé C Callegaro A Caubet M Fissette L. Inflammatory parameters associated with systemic reactogenicity following vaccination with adjuvanted hepatitis B vaccines in humans. Vaccine. (2019) 37:2004–15. doi: 10.1016/j.vaccine.2019.02.015 30850240

[46] de Jong SE Olin A Pulendran B. The impact of the microbiome on immunity to vaccination in humans. Cell Host Microbe. (2020) 28:169–79. doi: 10.1016/j.chom.2020.06.014 PMC7422826 32791110

[47] Tang B Tang L He W Jiang X Hu C Li Y. Correlation of gut microbiota and metabolic functions with the antibody response to the BBIBP-CorV vaccine. Cell Rep Med. (2022) 3:100752. doi: 10.1016/j.xcrm.2022.100752 36228621 PMC9589008

[48] Varga TV Niss K Estampador AC Collin CB Moseley PL. Association is not prediction: A landscape of confused reporting in diabetes - A systematic review. Diabetes Res Clin Pract. (2020) 170:108497. doi: 10.1016/j.diabres.2020.108497 33068662

