# Supplementary material for: An innate immune signature induced by AS01- or AS03-adjuvanted vaccines predicts the antibody response magnitude and quality consistently over time
Source: Front Immunol. 2024 Aug 14;15:1412732. doi: 10.3389/fimmu.2024.1412732 (PMC11349632; doi:10.3389/fimmu.2024.1412732)
Supplement: Supplementary file 1 [file Datasheet1.docx]

Supplementary Material

An innate immune signature induced by AS01- or AS03-adjuvanted vaccines predicts the antibody response magnitude and quality consistently over time

Setareh Tasdighian, Viviane Bechtold, Ahmed Essaghir, Yvan Saeys, Wivine Burny*

*** Correspondence:** Corresponding Author: Wivine Burny (WIVINE.BURNY@GSK.COM)

# Supplementary Figures

**
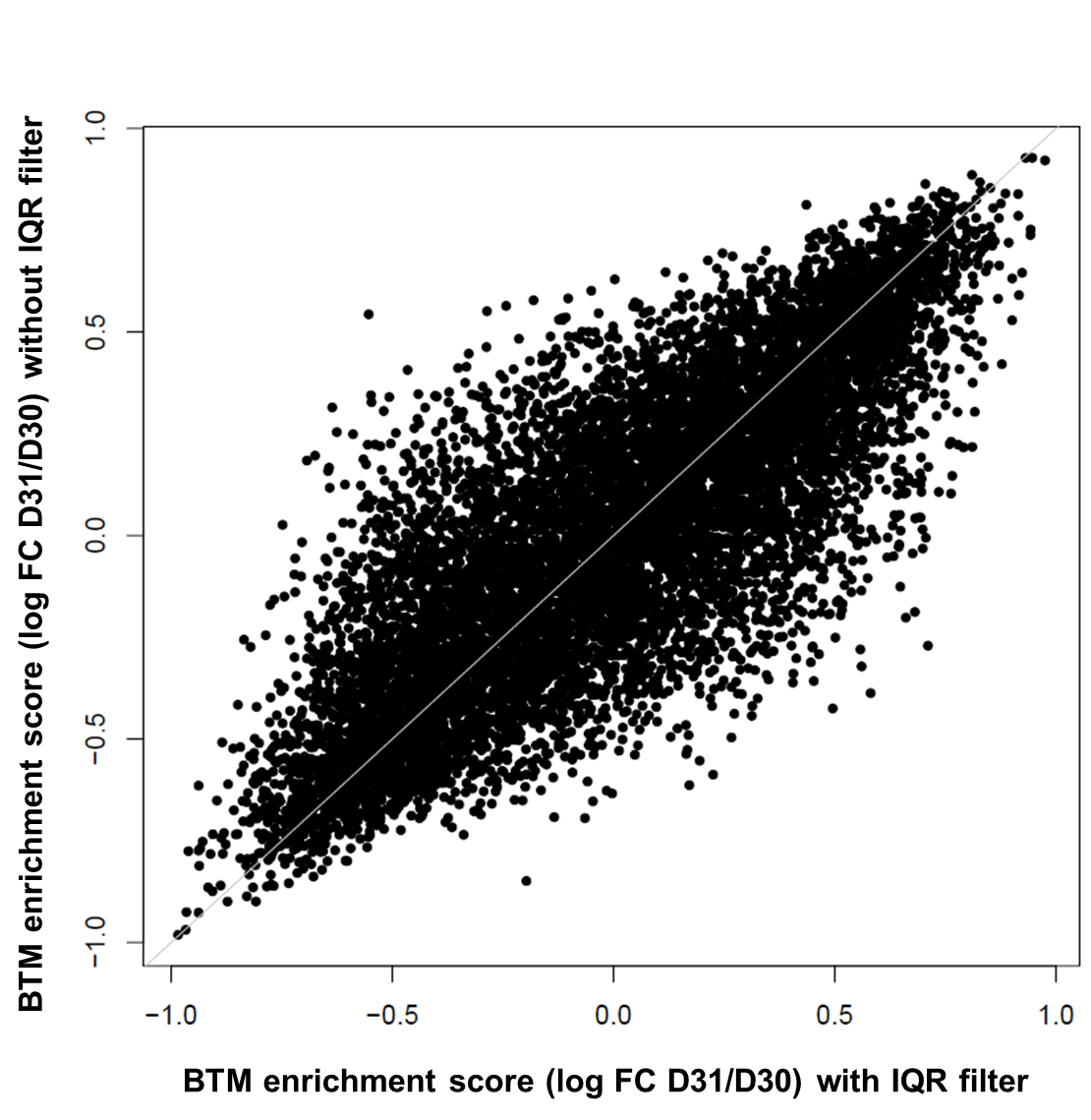
**

**Figure S1. Alignment between the BTM enrichment datasets obtained with or without IQR filtering.** Data cleaning involved application of a filter to eliminate probesets with an interquartile range (IQR) ≤ 0.75. The scatter plot presents the expression values (log fold-change [FC] of Day [D31] over D30) of genes included in the 103 BTMs that were shared between the datasets obtained with (x‑axis) or without (y-axis) the IQR filtering. BTM, blood transcriptional module. IQR, interquartile range.

**
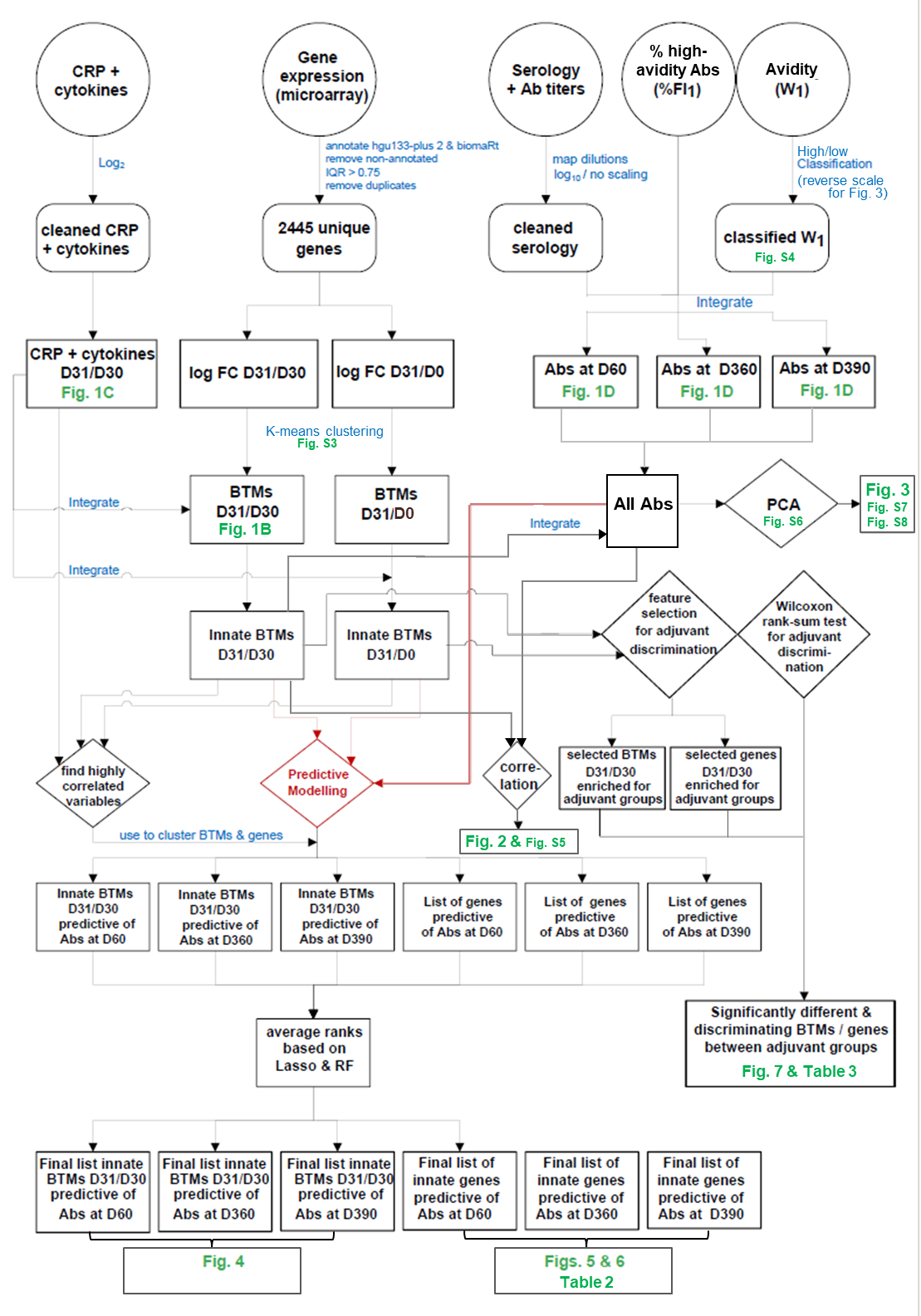
**

**Figure S2. Analysis pipelines.** CRP, C-reactive protein. D, day. IQR, interquartile range. FC, fold-change. BTM, blood transcriptional module described by Li et al. (30). Ab/Abs, antibody/antibodies. RF, Random Forest. PCA, principal component analysis. Figures and main tables are highlighted in green font.


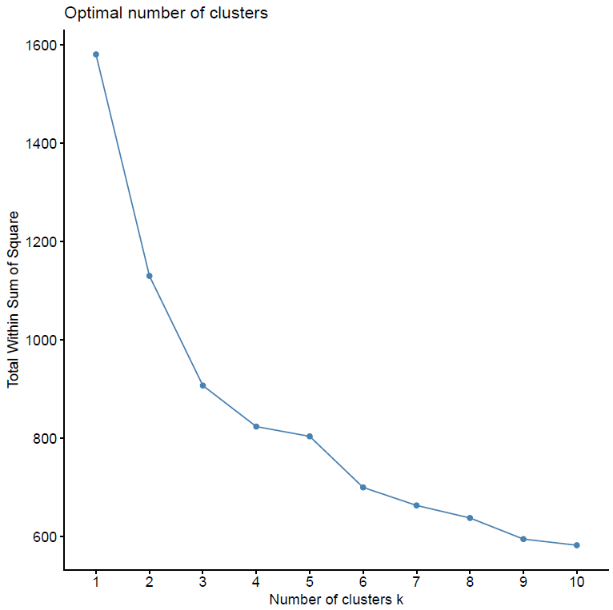


**Figure S3. K-means elbow plot**. The optimal number of functional blood transcriptional modules (BTMs) enriched by the log fold-change Day 31/Day 30 gene expression values was defined using K-means clustering according to the elbow method. The graph shows the total within sum of squares values (Y-axis) plotted against the number of clusters (k; X-axis). Based on this plot, and for consistency with previous analyses (29), the optimal number of clusters in the analyses was set at 5.

**
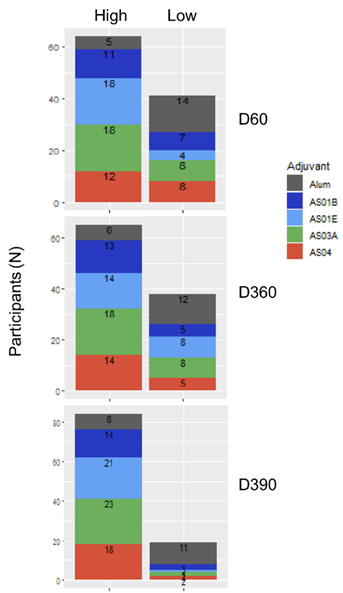
**

**Figure S4. W1 categories by adjuvant.** Stacked bar charts represent the distributions of participants assigned to the lower- and higher-avidity (W1) categories at days (D)60, D360 and D390, with the number of participants per adjuvant group indicated in the bar sections (color-coded as presented in the key).

**
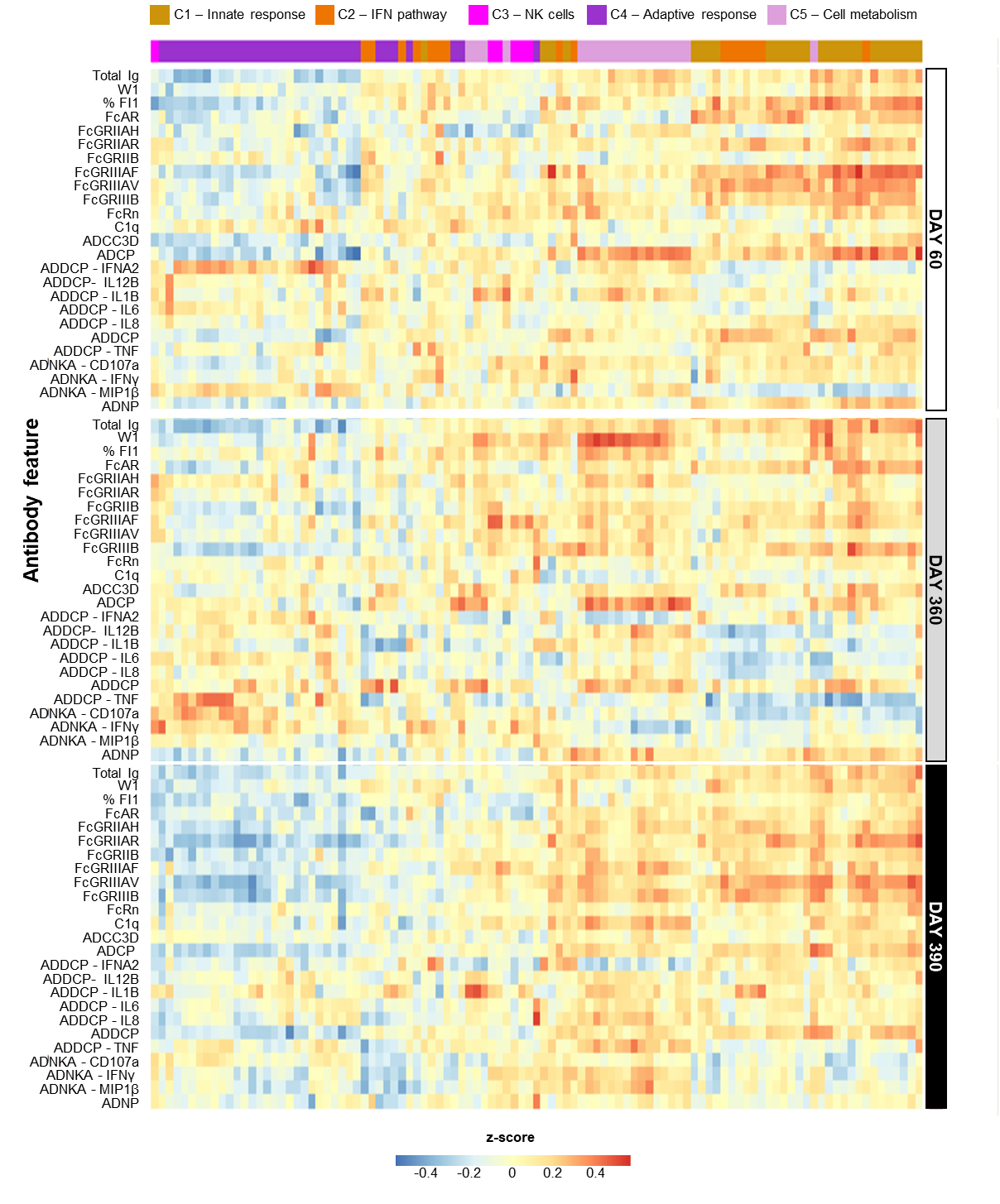
**

**Figure S5. Few correlations between the innate response and antibody features for AS04 and Alum.** Heatmap presents the Pearson correlation between blood transcriptional models (BTMs) and the quantitative and qualitative antibody (Ab) response parameters elicited by the study vaccines adjuvanted with AS04 or Alum (see Figure 2 for AS01 and AS03 data). BTMs (columns) are presented according to their data-driven clustering into the five functional BTM clusters (C1–C5) color-coded as presented in the key, and antibody response features (rows; see footnote of Table 1 for details) are presented by timepoint as depicted right of the heatmap.

**
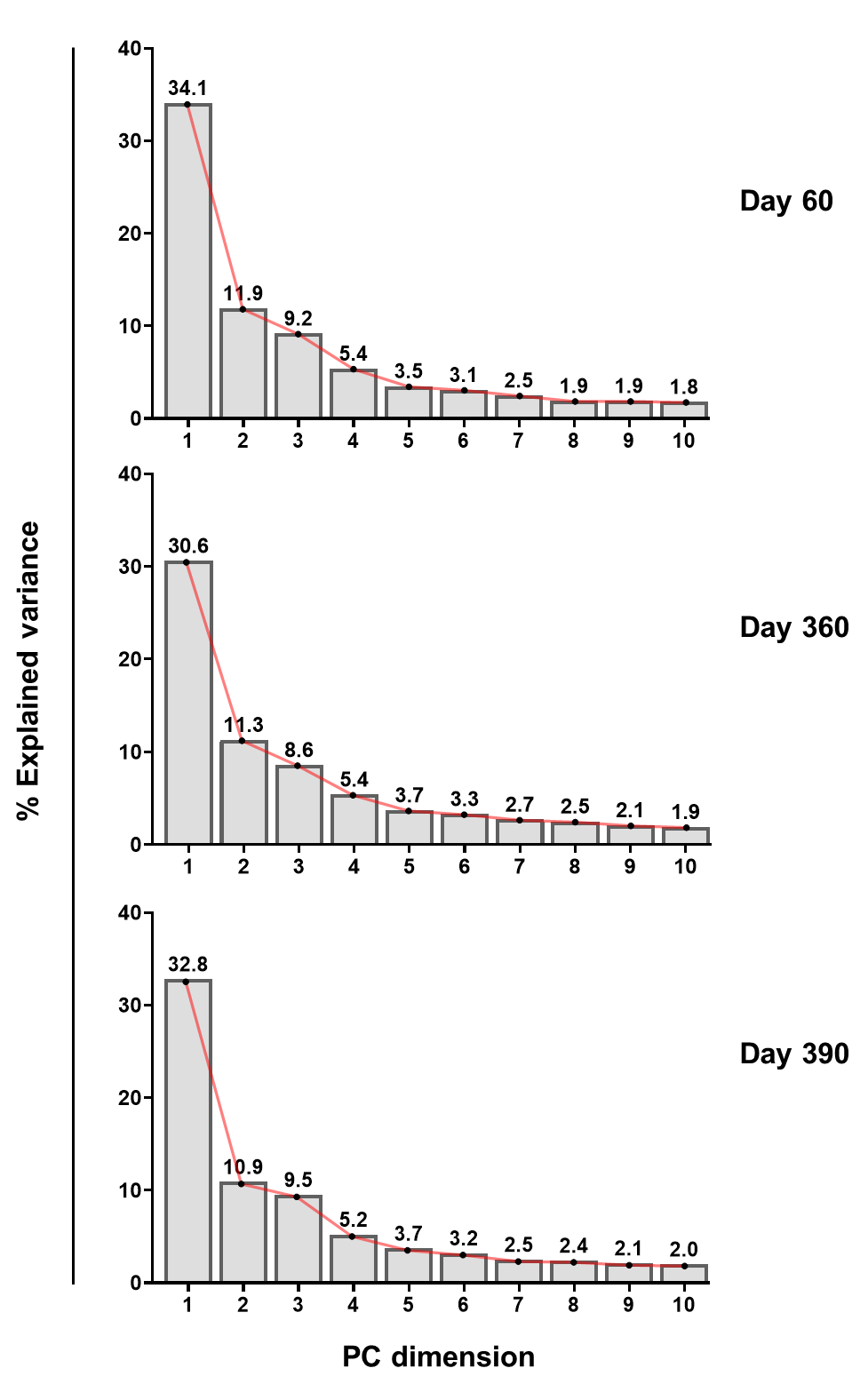
**

**Figure S6. Explained variances of the principal component analysis.** Scree plots show the variance explained by the first 10 principal components (PCs) from analyses of the innate response (in blood transcriptional models; BTMs) and the quantitative and qualitative antibody response features measured on day (D)60 (top), D360 (middle) and D390 (bottom) as shown in the scatter plots in Figure 3A.

**
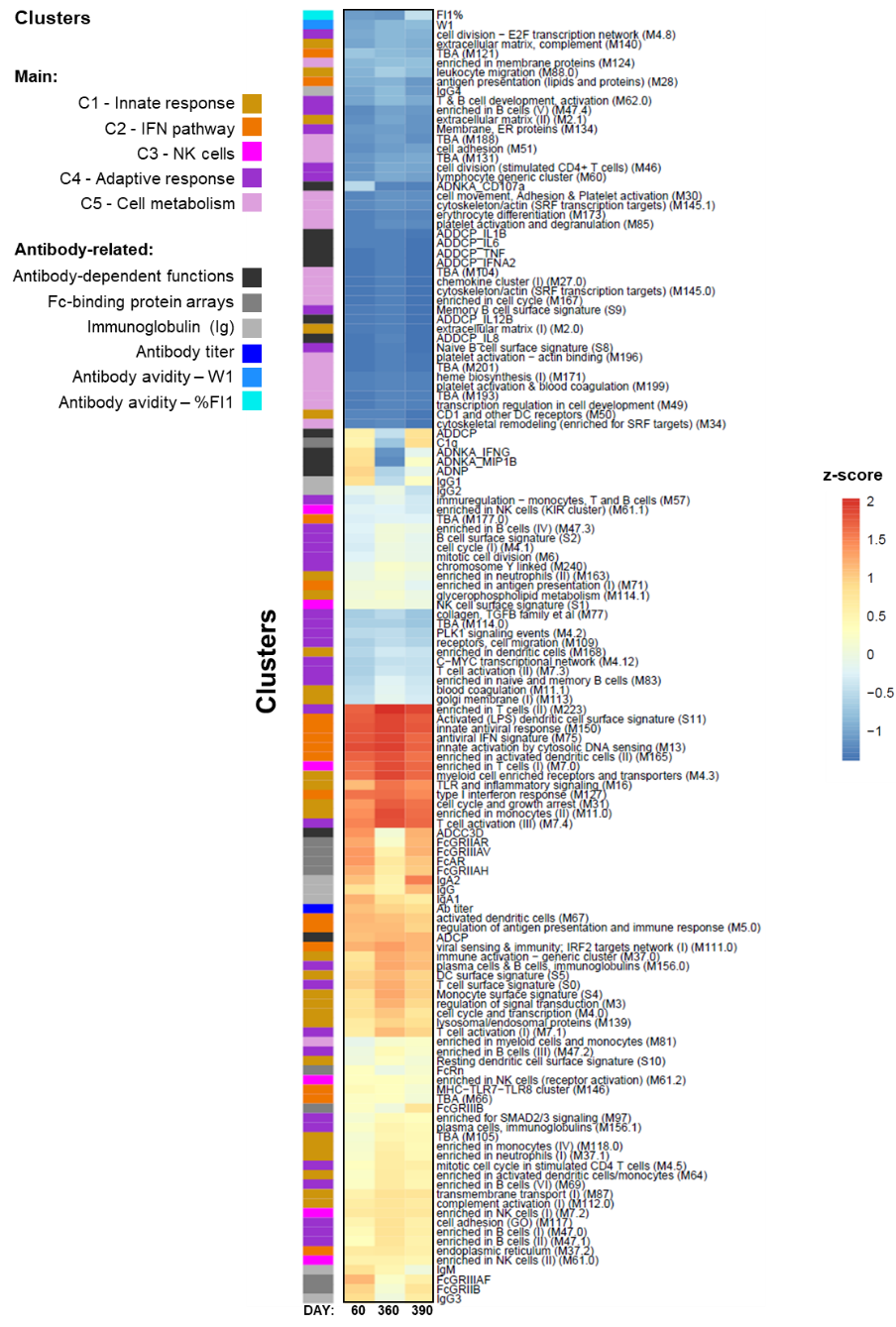
**

**Fig. S7. Contributions to the first principal component capturing the association between innate and adaptive response features.** Heatmap represents the contributions of the functional blood transcriptional models (BTM) clusters or antibody-related features (color-coded as presented in the key) to the first principal component (PC1) as shown in Figure 3B (note that W1 values were multiplied by -1 such that higher values correspond to higher avidity). Antibody-dependent functions include: ADCC3D, ADCP, ADDCP, ADNP, ADDCP_IFNA2, ADDCP_IL12B, ADDCP_IL1B, ADDCP_IL6, ADDCP_IL8, ADDCP_TNF, ADNK_CD107a, ADNK_IFNG, and ADNK_MIP1B; Fc-binding protein arrays include: FcGRIIAH, FcGRIIAR, FcGRIIB, FcGRIIIAF, FcGRIIIAV, FcGRIIIB, FcRn, FcAR, and C1q (see footnote of Table 1 for explanations).

**
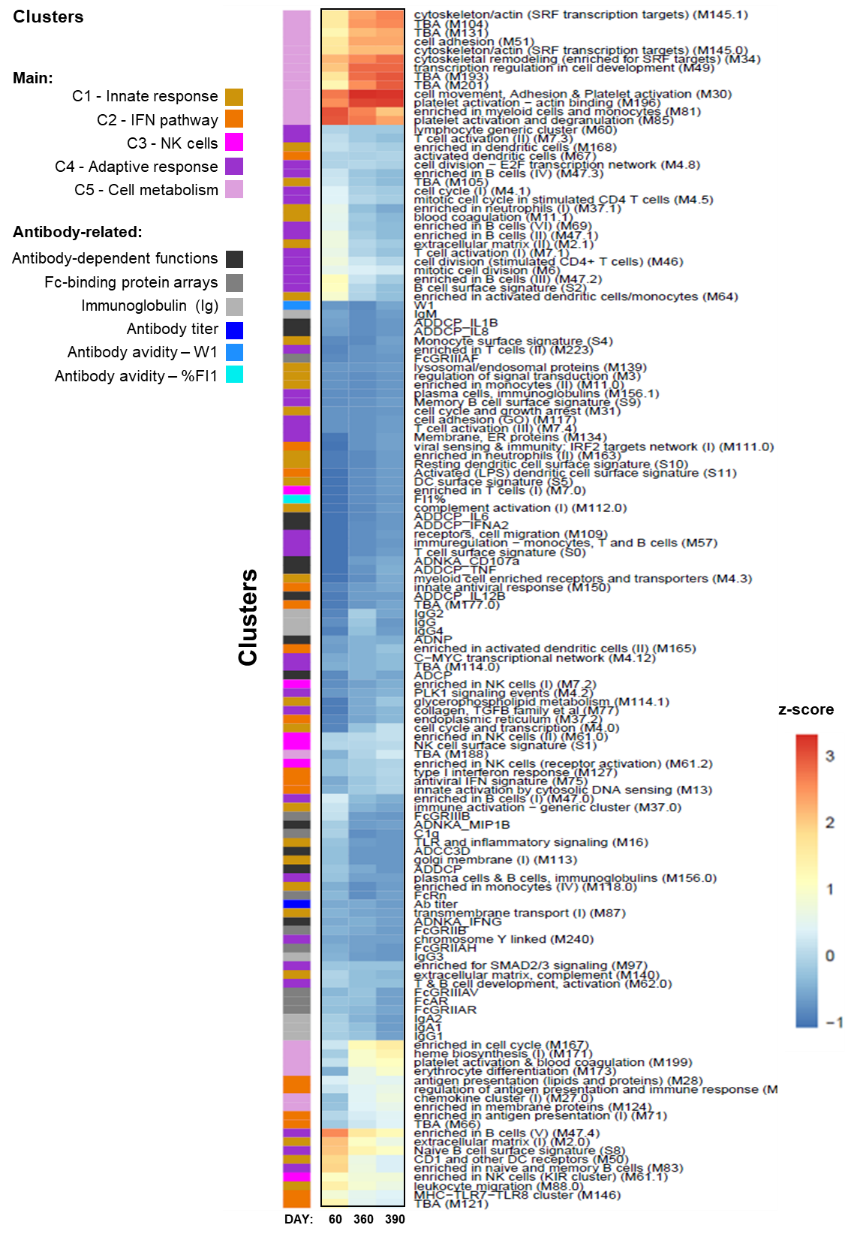
**

**Figure S8. Contributions to the second principal component capturing the association between innate and adaptive response features.** Heatmap represents the contributions of the functional blood transcriptional models (BTM) clusters or antibody-related features (color-coded as presented in the key) to the second principal component (PC2) as shown in Figure 3B (note that W1 values were multiplied by -1 such that higher values correspond to higher avidity). Antibody-dependent functions include: ADCC3D, ADCP, ADDCP, ADNP, ADDCP_IFNA2, ADDCP_IL12B, ADDCP_IL1B, ADDCP_IL6, ADDCP_IL8, ADDCP_TNF, ADNK_CD107a, ADNK_IFNG, and ADNK_MIP1B; Fc-binding protein arrays include: FcGRIIAH, FcGRIIAR, FcGRIIB, FcGRIIIAF, FcGRIIIAV, FcGRIIIB, FcRn, FcAR, and C1q (see footnote of Table 1 for explanations).

**
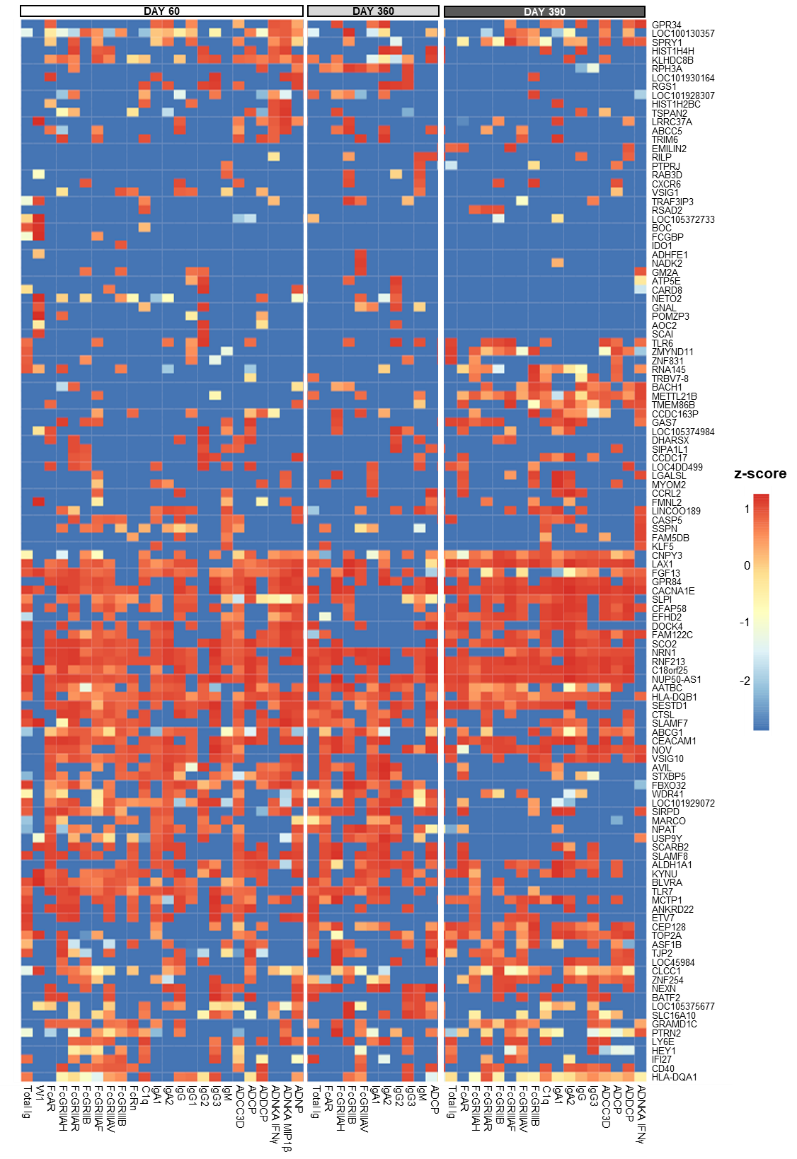
**

**Figure S9. Gene-based signature consistently predicts antibody features (top 10 selected features).** Heatmap of the union of the top 10 selected innate response features (including log fold-change D31/D30 serum cytokine and C-reactive protein [CRP] levels and gene expression values) that have the highest rank or predictive power to predict each antibody response feature on each day (day 60, 360 and 390) across the adjuvant groups (see Figure 5 for the top 5 selected features). Rankings derived by LASSO and Random Forest were averaged and standardized by antibody response feature. Tile colors correspond to the scaled ranks of the innate response, where red/blue corresponds to higher/lower predictive power (rather than to the direction of gene regulation). Row annotations represent the gene names.


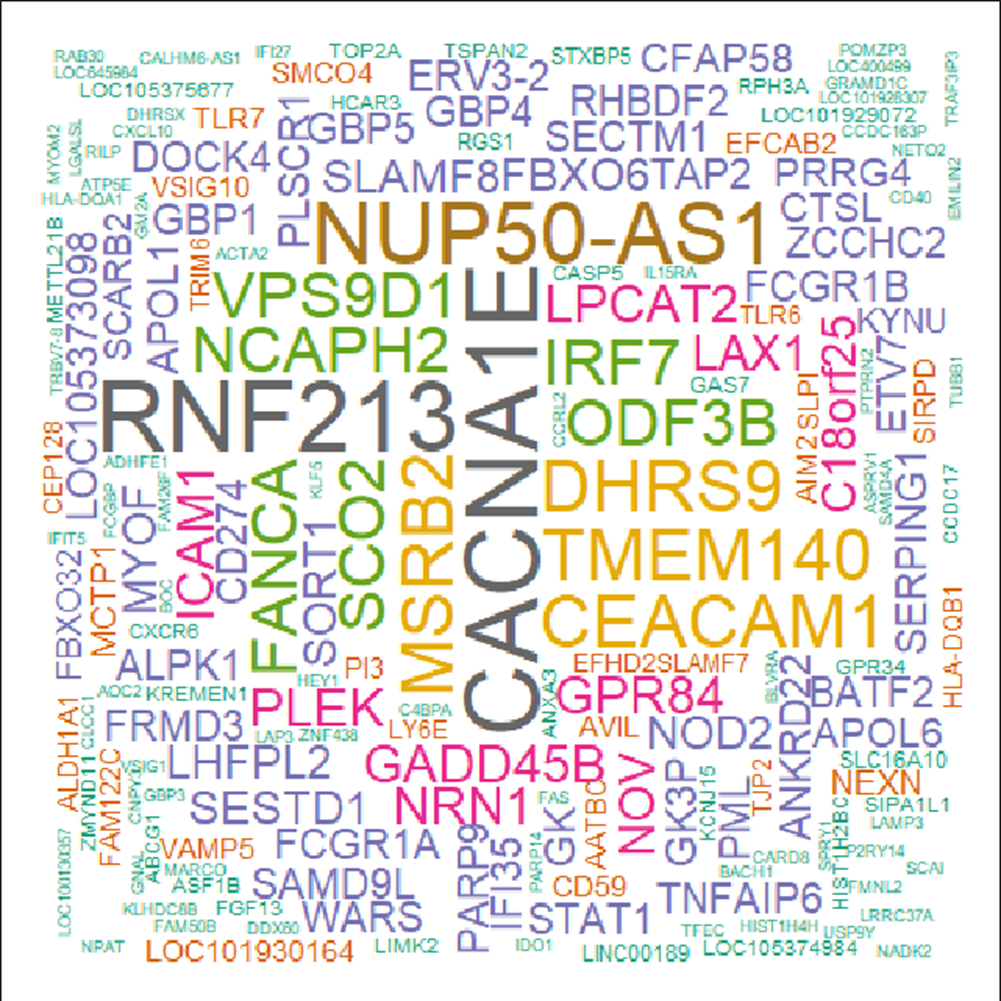


**Figure S10. Genes predictive of the longitudinal antibody response (top 10 selected features).** The word cloud represents the union of the top 10 genes able to predict the longitudinal antibody response (see Figure 6 for the word cloud related to the top 5 genes). Data were derived from all participants across all adjuvant groups and all timepoints among days 60, 360 and 390. The font size is proportional to the total number of predicted antibody response variables on a timepoint, with the specific timepoints detailed by gene in **Table S13** (second tab).
